# Nanostructure of PMMA/MAM Blends Prepared by Out-of-Equilibrium (Extrusion) and Near-Equilibrium (Casting) Self-Assembly and Their Nanocellular or Microcellular Structure Obtained from CO_2_ Foaming

**DOI:** 10.3390/nano11112834

**Published:** 2021-10-25

**Authors:** Suset Barroso-Solares, Victoria Bernardo, Daniel Cuadra-Rodriguez, Javier Pinto

**Affiliations:** 1 BioEcoUVA Research Institute on Bioeconomy, University of Valladolid, 47011 Valladolid, Spain; 2 Cellular Materials Laboratory (CellMat), Condensed Matter Physics Department, University of Valladolid, 47011 Valladolid, Spain; dcuadra@fmc.uva.es; 3 CellMat Technologies S.L., Paseo de Belen 9-A (CTTA Building), 47011 Valladolid, Spain; v.bernardo@cellmattechnologies.com

**Keywords:** block copolymers, CO_2_ gas dissolution foaming, heterogeneous nucleation, polymer foams

## Abstract

Blends of poly(methyl methacrylate) (PMMA) and a triblock copolymer poly(methyl methacrylate)-b-poly(butyl acrylate)-b-poly(methyl methacrylate) (MAM) have been obtained following both out-of-equilibrium (extrusion) and near-equilibrium (solvent casting) production routes. The self-assembly capability and the achievable nanostructures of these blends are analyzed by transmission electron microscopy (TEM) regarding their production route and potential for the achievement of nanocellular foams by CO_2_ gas dissolution foaming. The influence of the initial nanostructure of the solids on the obtained cellular structure of bulk and film samples is determined by high-resolution scanning electron microscopy (HRSEM) for diverse foaming conditions (saturation pressure, saturation temperature, and post-foaming stage), taking into account the required use of a foaming mold to achieve foams from films. Moreover, the influence of the nanostructuration on the presence of solid outer layers, typical of the selected foaming process, is addressed. Finally, consideration of a qualitative model and the obtained results in terms of nanostructuration, cellular structure, and foaming behavior, allow proposing a detailed cell nucleation, growth, and stabilization scheme for these materials, providing the first direct evidence of the cell nucleation happening inside the poly(butyl acrylate) phase in the PMMA/MAM blends.

## 1. Introduction

Enhancing energetic efficiency is a global technological priority in our society, being promoted by international organizations such as the European Union (EU) and the United Nations (UN) [1,2]. Decreasing CO_2_ emissions, which are directly related to energy consumption, is a key pillar for both organizations aiming to avoid, or lessen, present and future consequences of the global warming [1,2,3]. From a materials engineering point of view, several advances could significantly support this objective. Among other advances, decreasing the weight of diverse components employed in transportation would directly reduce the energy consumption of this sector (30.8% of the total energy consumption of the EU) [3,4]. Additionally, developing high-efficient thermal insulators would lower the energy consumption in household climatization (over half of the 27.2% of the total energy consumption of the EU) [3,5,6]. Some nanomaterials [7], such as nanocellular polymer foams have shown high potential to address both objectives [8,9,10]. The presence of a nanocellular structure inside the polymer provides significant weight reduction, while the mechanical properties of these materials are comparable to, or even better than, conventional foams [11,12,13]. In addition, the thermal insulation capability of nanocellular polymer foams could be significantly ameliorated due to the presence of the Knudsen effect on the nanometric cells, which hinders the thermal conduction through the gaseous phase [14]. Finally, the possibility to obtain such materials while keeping the transparency of the solid polymer precursor (e.g., polymethyl methacrylate (PMMA) [15], could lead to the development of windows with lower weight and significantly higher thermal insulation than conventional glass windows.

Currently, the most common production route of nanocellular polymer foams is the CO_2_ gas dissolution foaming process [16,17,18]. This is an eco-friendly environmental technique, which does not require the use of toxic solvents [16,17]. Generally, the gas is dissolved into a solid polymer precursor using a pressure vessel at controlled pressure and temperature. Then, once the polymer is fully saturated with the gas, thermal instability is generated to induce the polymer–gas phase separation, forming cell nuclei which later could grow into cells [16,17]. The nucleation process (i.e., the generation of cell nuclei) is a key step in the production of nanocellular polymer foams, being necessary to reach cell nucleation densities over 10^14^ nuclei per cubic centimeter of the solid precursor. In a simple approach, this nucleation process is regulated by the energy barrier that the nuclei should overcome to grow into a cell, also known as the Gibbs free energy barrier [19,20,21]. Accordingly, lowering the Gibbs free energy barrier would induce higher nucleation densities, as more nuclei will be stable and capable of growing instead of being reabsorbed into the polymer. The decrease in this energy barrier can be achieved by two main approaches. On one hand, increasing the amount of gas dissolved into the polymer by increasing the saturation pressure (P_sat_) and, sometimes, decreasing the saturation temperature (T_sat_) (e.g., PMMA), can provide cell nucleation densities as high as 10^16^ nuclei/cm^3^ in PMMA (P_sat_ = 20 MPa and T_sat_ = −32 °C) [22]. On the other hand, the introduction of an additional phase into the solid polymer precursor, generally nanoparticles or other polymers, can promote the cell nucleation by providing lower energy barriers usually located in the interfaces between phases [23]. The last approach, known as heterogeneous nucleation, is less dependent on the foaming parameters, being able to provide nanocellular foams under mild conditions [19,23,24,25].

Among other heterogeneous nucleating agents, block copolymers’ capability to generate self-assembled nanostructures is known to provide a straightforward route to produce nanocellular foams by inducing heterogeneous nucleation [24,25,26,27,28]. In particular, nanostructures with higher CO_2_-affinity, lower surface tension in the presence of CO_2_, and preferably lower strength than the surrounding polymer have been found to be optimal with this aim [25,28]. The most studied polymer–block copolymer system in the production of nanocellular foams are the blends of PMMA with a poly(methyl methacrylate)-b-poly(butyl acrylate)-b-poly(methyl methacrylate) (MAM) block copolymer [24,25,28]. The dispersed nanodomains present in PMMA/MAM blends with MAM contents ranging from 0.1–20 wt.% have successfully led to nanocellular polymer foams with an extraordinary nucleation efficiency of about 1, even at quite low-demanding foaming parameters such as 6 MPa and room temperature [24]. Moreover, the ability to produce a co-continuous nanostructure at a MAM content of about 75 wt.% allowed the obtaining of open cell nanocellular foams [29]. The versatility of these blends and their ability to provide nanocellular foams at mild processing conditions make them promising candidates for the future development of nanocellular foams by continuous extrusion foaming processes [10,30].

However, several questions about these materials have still not been answered. It is known that the PMMA/MAM nanostructures obtained from extrusion or injection processes are achieved by an out-of-equilibrium self-assembly process [31]. In particular, in the case of dispersed nanodomains, the extrusion parameters and viscosities of the polymers seem to play a relevant role in the achievable size and dispersion of the nanodomains [31]. Nevertheless, the equivalent near-equilibrium self-assembled structures for both low and high MAM content PMMA/MAM blends, and their effect on the resulting foam morphology have not been studied in detail. This deserves attention and in-depth study, as an excessive dependence of the nanostructures on processing conditions could hinder their use in continuous processes. In the last decade, the diverse self-assembly behavior of block copolymers close to equilibrium and out of equilibrium has been intensively studied [32,33]. For instance, Ronen et al. [32], proved that polystyrene-b-poly(4-vinylpyridine) (PS-b-P4VP) self-assembled out of equilibrium shows a nanostructure with P4VP within the micelle core. On the contrary, close to the equilibrium, the core of the micelles is composed of PS blocks, these changes being relevant to the development of nanoporous membranes.

Near equilibrium self-assembled MAM copolymers have been previously obtained as thin films by solvent casting, this approach being suitable for the study of PMMA/MAM blends [34]. Nevertheless, scarce results can be found in the literature about the CO_2_ gas dissolution foaming of polymer films, generally involving the use of metallic plates as gas diffusion barriers [35,36], or stretching-assisted foaming processes [37]. In fact, the CO_2_ gas dissolution foaming on thin materials presents severe limitations, as the foamed materials by this technique usually present solid outer layers with thicknesses of tens or even hundreds of microns [38]. On the contrary, PMMA/MAM blends could be a promising system to produce foamed films, as the heterogeneous cell nucleation induced by their nanostructure was previously reported to decrease the thickness of these solid layers in bulk samples [39]. It should be noticed that the foaming of PMMA/MAM films with a near-equilibrium nanostructure could be of particular interest in filtration processes. It is known that the co-continuous out-of-equilibrium nanostructure of 25/75 (weight ratio) PMMA/MAM blends led to open-cell nanocellular foams, which were proposed as potential filters [29]. Despite the open-cell structure, their production from bulk samples hindered that application, and it will be necessary to explore their obtention as films and the influence of the fabrication route on the co-continuous nanostructure.

In addition, it is widely accepted that the cell nucleation on PMMA/MAM happens on the nanodomains, and it was initially proposed that this process could happen on the interphases of these nanodomains [28]. Recently, theoretical considerations proposed to take into account not only the surface tension but also the line tension of a triphasic system (polymer matrix-dispersed phase-CO_2_) [23]. This approach has shown that the nucleation could happen even more readily completely inside the poly(butyl acrylate) (PBA) phase of the nanodomains, which could be the reason for the extraordinary nucleation efficiency of this system [23]. Although there is extensive evidence of the relationship between the nanodomains and the nucleation process [25,28], as well as accepted theoretical indications of the role of the PBA phase [23], there is no direct evidence about the nucleation process happening preferably in the PBA phase.

In this study, 90/10 and 25/75 PMMA/MAM blends films were obtained by solvent casting, and their near-equilibrium self-assembled nanostructure was studied and compared with the out-of-equilibrium nanostructure previously reported on bulk samples. Then, the foaming behavior of the near-equilibrium nanostructures was analyzed and compared with those of bulk samples presenting out-of-equilibrium nanostructures, aiming to understand the stability of these nanostructures, regarding their production route, and their potential use for continuous foaming processes. As the foaming behavior of films and bulk samples could differ, neat PMMA films and bulk samples foamed under the same conditions are taken as a reference, highlighting the impact of PMMA/MAM blends on the solid outer layers. Finally, the particularities of the near-equilibrium nanostructures of 90/10 PMMA/MAM blends provide the first direct information about the cell nucleation on the PBA phases, giving a clear picture of the process and potential explanations for the different foaming behavior between 90/10 and 25/75 PMMA/MAM blends.

## 2. Materials and Methods

### 2.1. Materials

Poly(methyl methacrylate) (PMMA) V825T (M_n_ = 43 kg/mol, M_w_ = 83 kg/mol, density (ρ) about 1.19 g/cm^3^, and glass transition temperature (T_g_) of 114.5 °C) was kindly supplied in the form of pellets by ALTUGLAS^®^ International (Colombes, France). The block copolymer poly(methyl methacrylate)-b-poly(butyl acrylate)-b-poly(methyl methacrylate) (MAM) grade M42 (36 wt.% of PBA, M_n_ = 85 kg/mol, M_w_ = 180 kg/mol, M_n_^PMMA block^ = 27 kg/mol, ρ = 1.08 g/cm^3^, T_g_^PMMA block^ = 107 °C and T_g_^PBA block^ = −42 °C) was kindly supplied also in the form of pellets by Arkema Company (Colombes, France). Chloroform (CHCl_3_) purchased from Sigma Aldrich (Madrid, Spain) was employed as solvent. Medical grade CO_2_ (99.9% purity) was used as blowing agent in the foaming experiments.

### 2.2. Preparation of Bulk and Film Solid Samples

For the bulk sample preparation, PMMA/MAM blends with weight ratios of 90/10 and 25/75 were produced in the form of pellets by extrusion using a Scamex CE02 single screw extruder (*L*/*D* = 28, *d* = 45 mm) with a temperature profile from 165–225° and screw speed of 60 rpm [29]. Solid precursors of the blends and neat PMMA were obtained by compression molding using a hot plate press provided by Remtex (Viladecavalls, Spain). Circular sheets with diameter of 15 cm and thickness of 2 mm were obtained by heating the pellets at 250 °C for 10 min without pressure, applying 2.2 MPa of pressure for 1 min at the same temperature, and then cooling the samples for 4 min at 2.2 MPa of pressure. Bulk samples of about 20 × 20 × 2 mm^3^ were finally prepared for the foaming tests.

In the case of the film samples, neat PMMA and 90/10 and 25/75 PMMA/MAM blends films were obtained by solvent casting. For each case, the proper amount of PMMA and MAM pellets were simultaneously dissolved at 20 wt.% in CHCl_3_ at 50 °C and using magnetic stirring. Then, about 5 mL of the solution were cast in a circular Teflon mold (38 mm diameter × 5.5 mm depth) and left to dry under an aspiration hood for at least 24 h. Then, the dry films were removed from the mold and further dried for 6 h under vacuum at room temperature. Finally, samples of about 20 mm length, 4 mm width, and 75 µm thickness were cut for the foaming tests.

### 2.3. Preparation of Bulk and Film Foamed Samples

The gas dissolution foaming experiments were carried out in a high-pressure vessel (model PARR 4681, Parr Instrument Company, Moline, IL, USA) at saturation temperatures ranging from 40–60 °C, saturation pressures of 20 or 30 MPa. These foaming parameters were selected to generally allow the foaming of both bulk and film samples, as well as to avoid the degeneration of the nanocellular structure of 25/75 PMMA/MAM blends, according to previous results [29,35]. For all the foaming tests, the samples were kept in the pressure vessel for 24 h to ensure the complete CO_2_ saturation of the polymer. Once the saturation step was concluded, the pressure was quickly reduced (about 100 MPa/s of pressure drop peak at the first instants and reaching ambient pressure after 30–35 s), and the samples were extracted from the pressure vessel. Some samples were left to stabilize at room temperature, whereas other samples were subjected to a post-foaming step by immersion for 1 min in a heated water bath at 40, 60, or 80 °C. The foaming of the film samples was achieved by using a specific mold throughout the whole foaming process that allows the simultaneous foaming of several samples, while restricts the gas diffusion through the faces of the films. Details about this setup can be found elsewhere [35]. It should be noted that the films produced in this work were not able to foam without the use of this mold at the employed foaming parameters.

### 2.4. Characterization Techniques

The nanostructuration of the solid PMMA/MAM films was studied by transmission electron microscopy (TEM) with a Jeol JEM 1011 (Jeol, Tokyo, Japan) electron microscope (Electron Microscopy Lab.–Nanochemistry Dept., Instituto Italiano di Tecnologia). Each sample was cut under cryogenic conditions (−60 °C), in about 80–90 nm thin slices using a Leica EM UC6 Ultramicrotome equipped with a cryo-system Leica EM FC6 and a specific diamond knife for low temperature. The obtained slices were collected onto a 200 mesh formvar/carbon-coated copper TEM grid.

TEM micrographs were analyzed using the software ImageJ/FIJI [40], determining the average micelle size or lamella thickness by measuring more than 100 nanodomains of different areas for each sample. Moreover, in the case of dispersed nanodomains (i.e., micelles) the volumetric micelle density (*N_n_*, number of micelles per cm^3^ of the solid film) was obtained from the number of micelles in the TEM micrographs (*n*) and their areas (*A*) using Equation (1) [28].

*N_n_* = (*n*/*A*)^3/2^ (1)

The density of bulk foamed samples was determined after removing the solid outer layers by the water-displacement method, carrying out at least three measurements for each sample. Then, the reported average values and standard deviation (SD) of the relative density were obtained from the ratio between the measured densities and the density of the solid matrix. The density of foamed films is not reported, as the impossibility to remove the solid outer layers without damaging the sample makes the obtained values misleading and not representative of their cellular structure.

Finally, the cellular structure of both bulk and film foamed samples was analyzed by high resolution scanning electron microscopy (HRSEM, model Quanta 200FED, FEI, Hillsboro, OR, USA). The samples were frozen in liquid nitrogen and fractured to expose their cellular structure. Then, these exposed surfaces were coated with gold (sputter coater model SCD 004, Balzers Union, Madrid, Spain). Average values and standard deviation (SD) of cell size and solid skin thickness were determined using a specific software based on ImageJ/FIJI by measuring at least 100 cells or 10 thicknesses of the solid skin, respectively [40,41].

## 3. Results and Discussion

### 3.1. Out of Equilibrium and Near Equilibrium Nanostructuration of PMMA/MAM Blends

**Figure 1:**
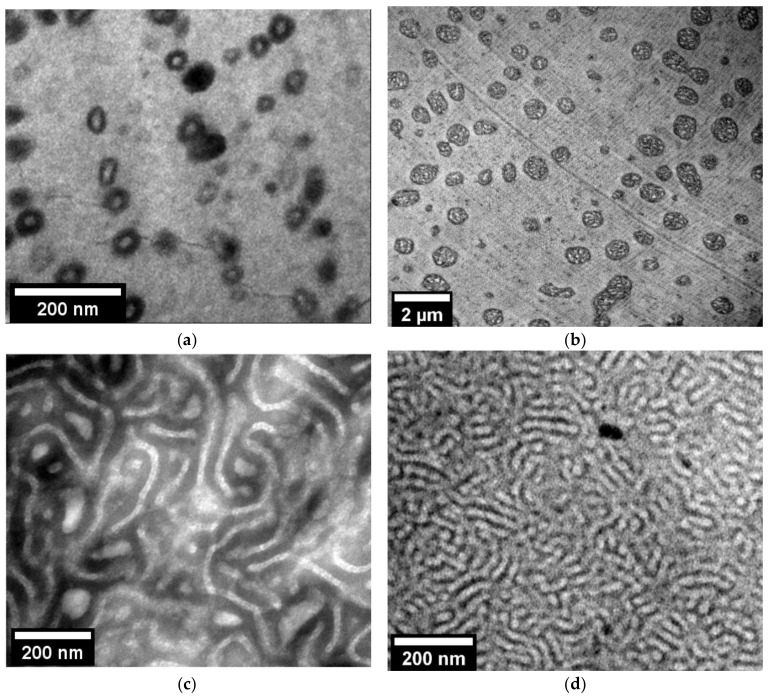
TEM micrographs of the nanostructuration of (**a**) 90/10 PMMA/MAM out-of-equilibrium blend (Reprinted from Chemical Engineering Journal, 243, J. Pinto, M. Dumon, M. Pedros, J. Reglero, M.A. Rodriguez-Perez, Nanocellular CO_2_ foaming of PMMA assisted by block copolymer nanostructuration, Pages 428–435, Copyright (2014) [28], with permission from Elsevier); (**b**) 90/10 PMMA/MAM near-equilibrium blend, please notice the above one-order-of-magnitude difference between scales; (**c**) 25/75 PMMA/MAM out-of-equilibrium blend (Reprinted with permission from The Journal of Physical Chemistry C, 118, 9. Block Copolymers Self-Assembly Allows Obtaining Tunable Micro or Nanoporous Membranes or Depth Filters Based on PMMA; Fabrication Method and Nanostructures, J. Pinto, M. Dumon, M.A. Rodriguez-Perez, R. Garcia, C. Dietz, Pages 4656–4663. Copyright (2014) American Chemical Society) [29]; (**d**) 25/75 PMMA/MAM near-equilibrium blend.

The out-of-equilibrium nanostructuration of 90/10 and 25/75 PMMA/MAM bulk blends obtained by extrusion (Figure 1) has been reported in previous works [28,29]. 90/10 PMMA/MAM bulk blends present dispersed micelles (Figure 1a), with an average size of about 30 nm and a volumetric micelle density (*N_n_*) of up to 10^14^ micelles/cm^3^ [28]. The near-equilibrium nanostructuration of 90/10 PMMA/MAM films obtained by solvent casting preserves a micellar-like dispersed nanostructuration but shows significant changes in size and volumetric density (Figure 1b). Near equilibrium 90/10 PMMA/MAM micelles present average diameters of about 560 nm (Figure S1, see Supplementary Materials), while their volumetric density (*N_n_*) accordingly decreases down to about 10^11^ micelles/cm^3^ (i.e., bigger micelles involve a higher number of MAM polymer chains in their formation, being inversely related to the number of micelles that can be formed) [42]. Taking into account the dominant role of the PMMA/MAM micelles on the cell nucleation, it cannot be expected to obtain nanocellular foams from near-equilibrium 90/10 PMMA/MAM blends (i.e., nucleation densities over 10^14^ are required) [16]. The strong dependence of the 90/10 PMMA/MAM nanostructuration with the fabrication conditions could be a drawback on the potential development of continuous foaming processes to obtain nanocellular foams by extrusion foaming. It would be necessary to employ not only appropriate production parameters to allow the continuous foaming process [30], but also to ensure an adequate out-of-equilibrium nanostructuration.

On the contrary, the differences between out-of-equilibrium and near-equilibrium 25/75 PMMA/MAM blends are less apparent (Figure 1c,d). 25/75 PMMA/MAM bulk blends present a co-continuous lamellar-like nanostructurarion (Figure 1c), with thicknesses of about 20–30 nm [29]. Whereas 25/75 PMMA/MAM films show a similar co-continuous lamellar-like nanostructuration, with slightly thinner features of about 10–20 nm (Figure 1d). Therefore, both out-of-equilibrium and near-equilibrium 25/75 PMMA/MAM blends are suitable to produce nanocellular foams, and the lower dependence of their nanostructuration on the fabrication conditions makes these blends suitable candidates for the development of continuous extrusion foaming processes.

In both cases, it should be taken into account that the nanostructuration could be modified during the CO_2_ saturation stage due to the increased mobility of the plasticized polymers chains. However, previous results proved that the nanostructuration of 90/10 PMMA/MAM blends suffers no significant changes regarding the average dimensions of the micelles, whereas 25/75 PMMA/MAM blends could present a slight increase in the lamellas thickness but remaining in the nanometric range [43].

### 3.2. Nanocellular Foams Obtained from Out-of-Equilibrium and Near-Equilibrium Nanostructuration of PMMA/MAM Blends

Samples of 90/10 and 25/75 PMMA/MAM films were subjected to the gas dissolution foaming procedure in order to check if the near-equilibrium nanostructurations behave as expected. In the first step, two different saturation pressures (20 and 30 MPa) and three different saturation temperatures (40, 50, and 60 °C) were employed. The samples were then stabilized at room temperature. As expected, the near-equilibrium 90/10 PMMA/MAM blends were not able to provide nanocellular structures independently of the foaming parameters (Figure S2, see Supplementary Materials), and therefore will not be further discussed until Section 3.4 (to determine the cell nucleation mechanisms on the nanostructuration).

Bulk neat PMMA and 25/75 PMMA/MAM blends, as well as neat PMMA films, were also foamed under the same conditions as the 25/75 PMMA/MAM films to be employed as reference samples (Table 1). Neat PMMA foams obtained at 20 MPa present microcellular structures, both for bulk and film samples, with average sizes about 1–2 µm (Table 1 and Figure S3, see Supplementary Materials) and high relative densities (about 0.8, measured on the bulk samples, see Table 1). The low expansion is particularly noticeable on neat PMMA film samples. In fact, the expansion limitation induced by the mold required to allow the foaming even avoids the foaming at the lower saturation temperature (40 °C) and allows only very low porosities at higher temperatures (Figure S3, see Supplementary Materials). On the contrary, 25/75 PMMA blends, both bulk and films, present nanocellular structures (Table 1 and Figure S4, see Supplementary Materials). On the one hand, bulk 25/75 PMMA/MAM blends present relative densities from 0.71–0.65, decreasing with the saturation temperature, and average cell sizes from 100–120 nm, increasing with the saturation temperature (Table 1). On the other hand, 25/75 PMMA/MAM films clearly show lower expansions related to the constraining induced by the mold, particularly at 40 °C (Figure S4b, see Supplementary Materials). Moreover, the average cell sizes range from 96 to 210 nm, increasing with the saturation temperature (Table 1). Additionally, some tendency towards coalescence of the cellular structures, leading to larger voids, can be observed in these samples (Figure S4). This is probably related to the expansion restriction imposed by the foaming mold, which induces a higher overpressure inside the foam (i.e., the foam cannot expand normally following this overpressure; thus, the sustained inner overpressure could induce coalescence).

**Figure 2:**
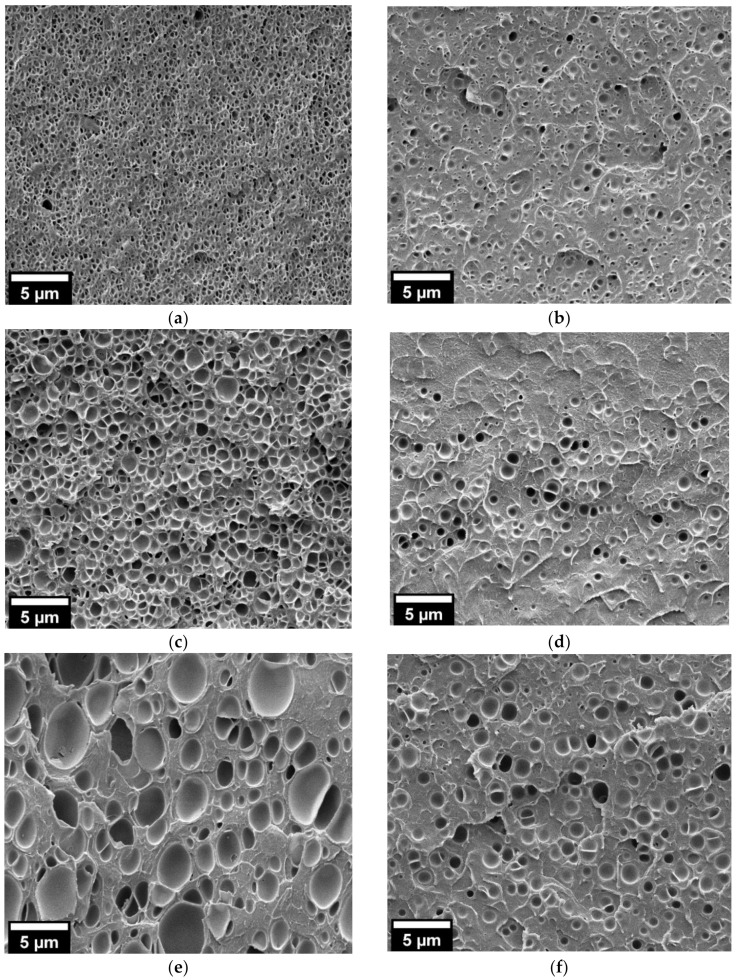
HRSEM micrographs of the cellular structure of the neat PMMA foams obtained from bulk samples (left) and films (right) at 30 MPa and different saturation temperatures (**a**,**b**) 40 °C; (**c**,**d**) 50 °C; (**e**,**f**) 60 °C.

**Figure 3:**
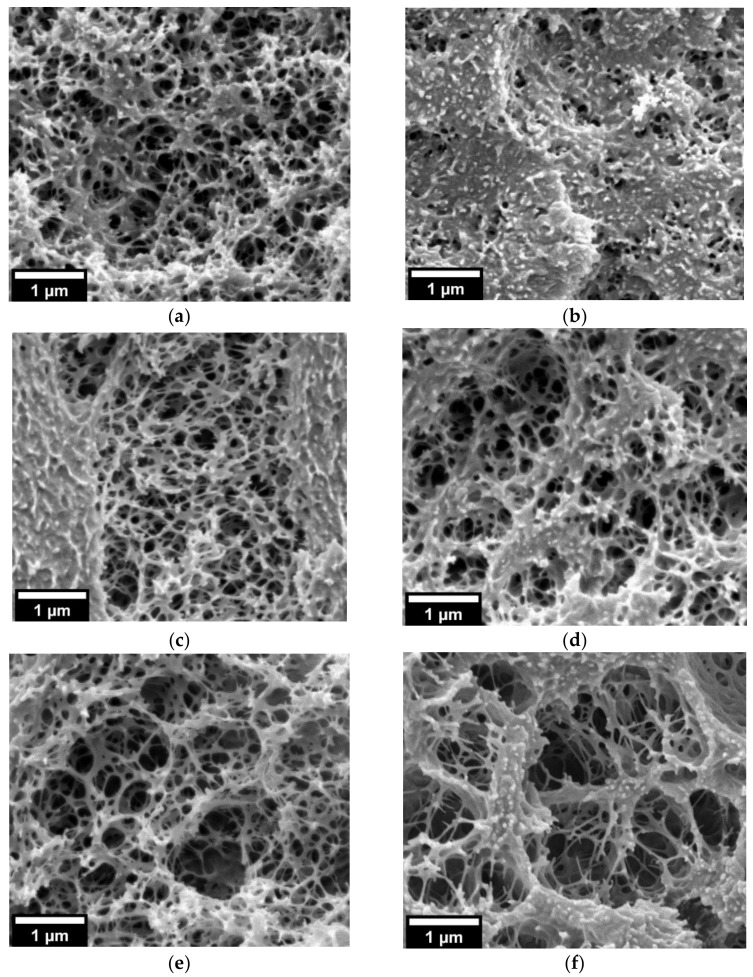
HRSEM micrographs of the cellular structure of the 25/75 PMMA/MAM foams obtained from bulk samples (left) and films (right) at 30 MPa and different saturation temperatures (**a**,**b**) 40 °C; (**c**,**d**) 50 °C; (**e**,**f**) 60 °C.

Higher saturation pressure (30 MPa) induces the presence of cellular structure on both neat PMMA and 25/75 PMMA/MAM films even at the lower saturation temperature (40 °C) (Figure 2 and Figure 3). Bulk neat PMMA samples achieve medium relative densities of about 0.62–0.67 and average cell sizes from 325 nm to 1.7 µm, increasing with the saturation temperature (Table 1 and Figure 2). Film neat PMMA samples again reach low porosities, as seen in the micrographs, while their average cell sizes range from 650 nm to 1.3 µm, also increasing with the saturation temperature (Table 1 and Figure 2). At this saturation pressure, 25/75 PMMA/MAM bulk and film samples present more similar cellular structures, except for samples obtained at a saturation temperature of 40 °C, which show significantly lower expansion when foamed in the form of film (Figure 3). Bulk 25/75 PMMA/MAM samples reach relative densities from 0.66 to 0.59, decreasing with the saturation temperature (Table 1). At the same time, the average cell size of bulk samples reaches about 120–140 nm for bulk samples and increases with temperature from about 95 to 260 nm for film samples (Table 1). As explained in the Materials and Methods section, the relative density of foamed films was not measured, as the thickness of the samples made it impossible to remove the solid outer layers to determine with accuracy the density of the foamed core. However, HRSEM micrographs of the cellular structure (Figure 3) allow estimating that both out-of-equilibrium and near-equilibrium nanostructuration of 25/75 PMMA/MAM blends have similar potential to reach medium–high porosities. As determined with the neat PMMA samples, lower porosities of the film samples are mainly related to the foaming mold required to foam these films. Moreover, the sustained overpressure inside the 25/75 PMMA/MAM films, constrained during the foaming by the mold, induces cell coalescence leading to the presence of micrometric voids clearly appreciable on low magnification HRSEM micrographs of the foamed films (Figure S5, see Supplementary Materials). Both undesirable effects could be avoided by developing new strategies to foam polymer films that overcome the expansion limitation induced by the mold required to foam these samples [35,36].

In addition, it should be highlighted that no evidence of a relationship between the nanostructure thicknesses and the cell size of the solid and foamed samples, respectively, was found. It could be expected that smaller nanostructure features, such as those observed in the samples fabricated near equilibrium could lead to smaller cell sizes, but the cell coalescence induced by the overpressure on the film samples hinders a direct comparison of the results.

As the density reductions achieved by both reference neat PMMA and 25/75 PMMA/MAM bulk and film samples were quite low, a post-foaming step was introduced on the foaming procedure. Taking into account the significant cell coalescence observed on 25/75 PMMA/MAM films samples foamed at 30 MPa and 60 °C (Figure S5, see Supplementary Materials), the saturation parameters for the two-step foaming tests were fixed at 30 MPa and 50 °C. After this saturation stage, all the samples were subjected to a post-foaming step in a heated bath for 1 min at temperatures ranging from 40–80 °C (Table 2).

**Figure 4:**
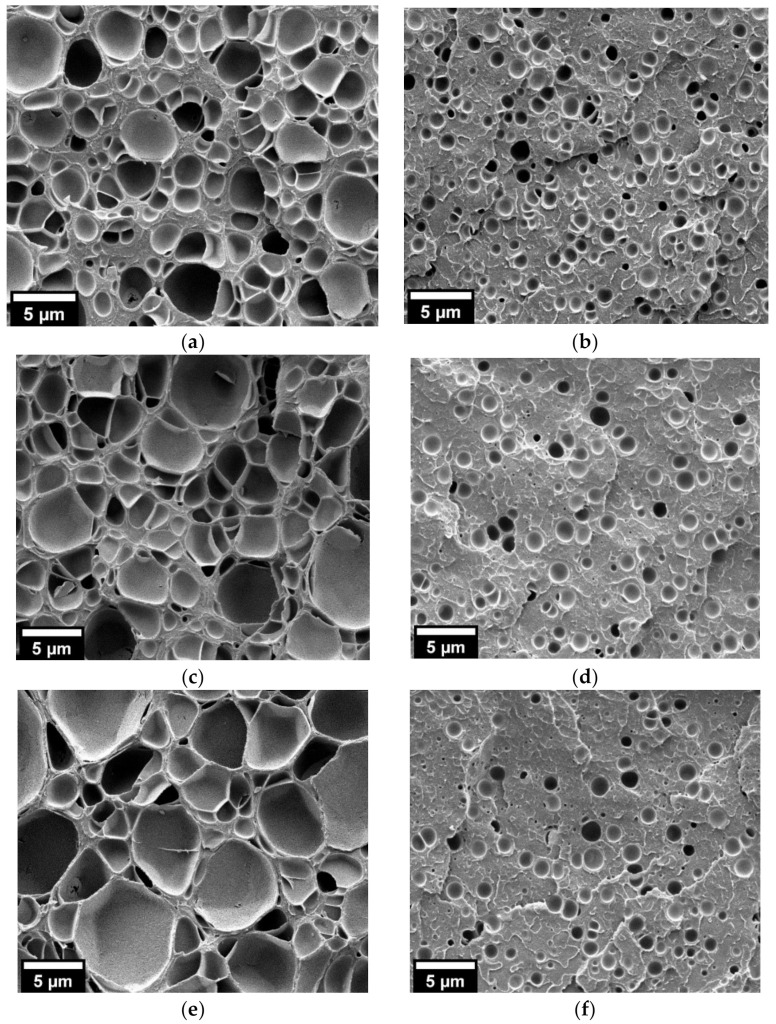
HRSEM micrographs of the cellular structure of the neat PMMA foams obtained from bulk samples (left) and films (right) at saturation pressure and temperature of 30 MPa and 50 °C, respectively, and a post-foaming procedure carried out for 1 min at different temperatures (**a**,**b**) 40 °C; (**c**,**d**) 60 °C; (**e**,**f**) 80 °C.

Bulk neat PMMA samples obtained following this procedure showed the expected behavior, with their relative densities decreasing from 0.67 to 0.26 and cell sizes increasing from 650 nm to 1.8 µm with the post-foaming temperature increase (Table 2 and Figure 4). On the contrary, neat PMMA foamed films showed little influence of the post-foaming procedure, with similar cellular structures and cell sizes of about 1.2–1.5 µm (Table 2 and Figure 4). Therefore, it was confirmed that the expansion constriction induced by the foaming mold plays a major role in the foam expansion, especially in two-step foaming processes.

**Figure 5:**
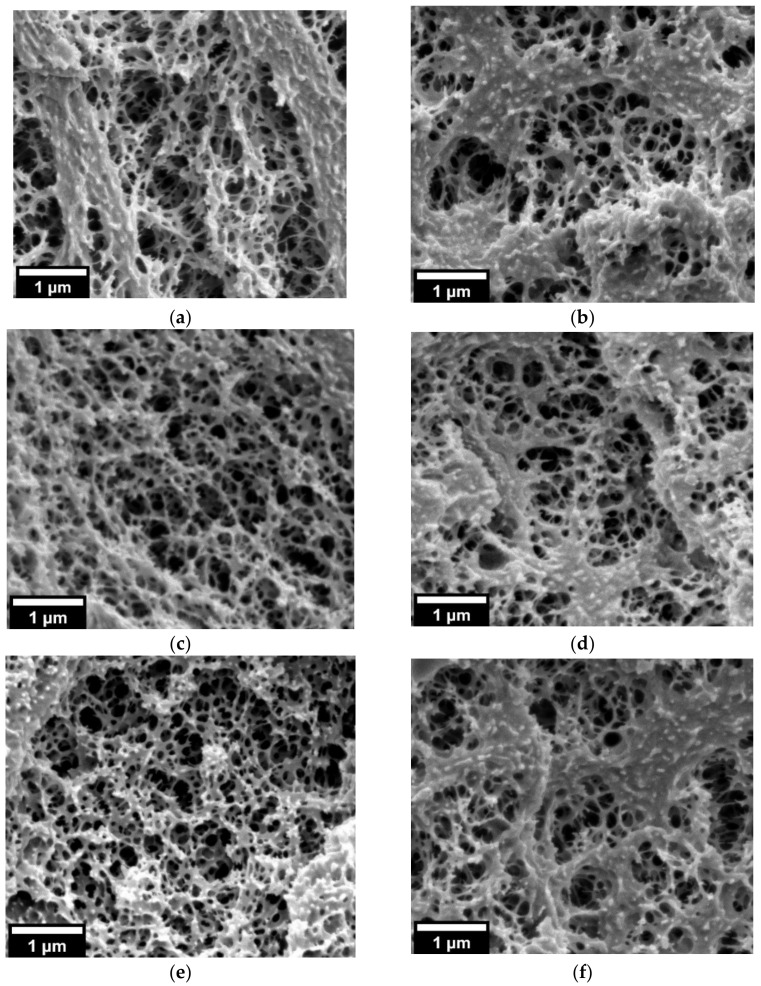
HRSEM micrographs of the cellular structure of the 25/75 PMMA/MAM foams obtained from bulk samples (left) and films (right) at saturation pressure and temperature of 30 MPa and 50 °C, respectively, and a post-foaming procedure carried out for 1 min at different temperatures (**a**,**b**) 40 °C; (**c**,**d**) 60 °C; (**e**,**f**) 80 °C.

In addition, 25/75 PMMA/MAM bulk samples were not affected by the post-foaming procedure, showing similar relative densities of about 0.68 and cell sizes of about 100–120 nm independently of the post-foaming parameters (Table 2 and Figure 5). The same behavior was found for 25/75 PMMA/MAM films, also showing slightly larger cell sizes than the bulk samples as previously explained. Additionally, the post-foaming procedure of 25/75 PMMA/MAM films had no influence on cell coalescence into micrometric voids (Figure S6, see Supplementary Materials).

This unexpected behavior, with the post-foaming process not affecting even the bulk samples or inducing cell degeneration, will be further discussed together with the cell nucleation in the last section.

### 3.3. Influence of the Co-Continuous Out-of-Equilibrium and Near-Equilibrium Nanostructuration of 25/75 PMMA/MAM Blends on the Solid Outer Layers

The removal of the solid outer skins of nanocellular foams produced by gas dissolution foaming, or even better, avoiding their formation during the foaming process, has been identified as a major challenge in the production of these materials [10,16]. On the one hand, the use of metallic plates to avoid gas diffusion through the faces of the samples, proposed by Siripurapu et al. [36], can decrease the thickness of these layers, allowing the foaming of films. On the other hand, the predominant role of the heterogeneous nucleation on PMMA/MAM blends with low MAM amounts (e.g., 10 wt.%) modifies the processes controlling the formation of these solid outer layers [39]. Accordingly, in order to understand if the combination of both approaches could provide additional reduction in the solid outer layers, the thickness of these layers was measured for all the neat PMMA and 25/75 PMMA/MAM bulk and film foamed samples (Table 1 and Table 2).

Neat PMMA bulk foamed samples obtained without post-foaming present thick solid outer layers, which decrease with the saturation temperature from about 270 to 240 µm and 180 to 90 µm for saturation pressures of 20 and 30 MPa, respectively (Table 1). On the contrary, neat PMMA foamed films show solid outer layers between 5 and 15 µm (Table 1), although the low porosity of these samples makes it difficult to determine with accuracy the extension of the solid layers. Accordingly, and as expected, the restriction of the gas diffusion in films due to the foaming mold has a strong influence on the solid outer layers’ thickness. 25/75 PMMA/MAM bulk and film foamed samples show a quite different behavior, with their solid outer layer thicknesses generally ranging from 3 to 10 µm, without clear dependence on the foaming parameters or even the use of the foaming mold with films.

**Figure 6:**
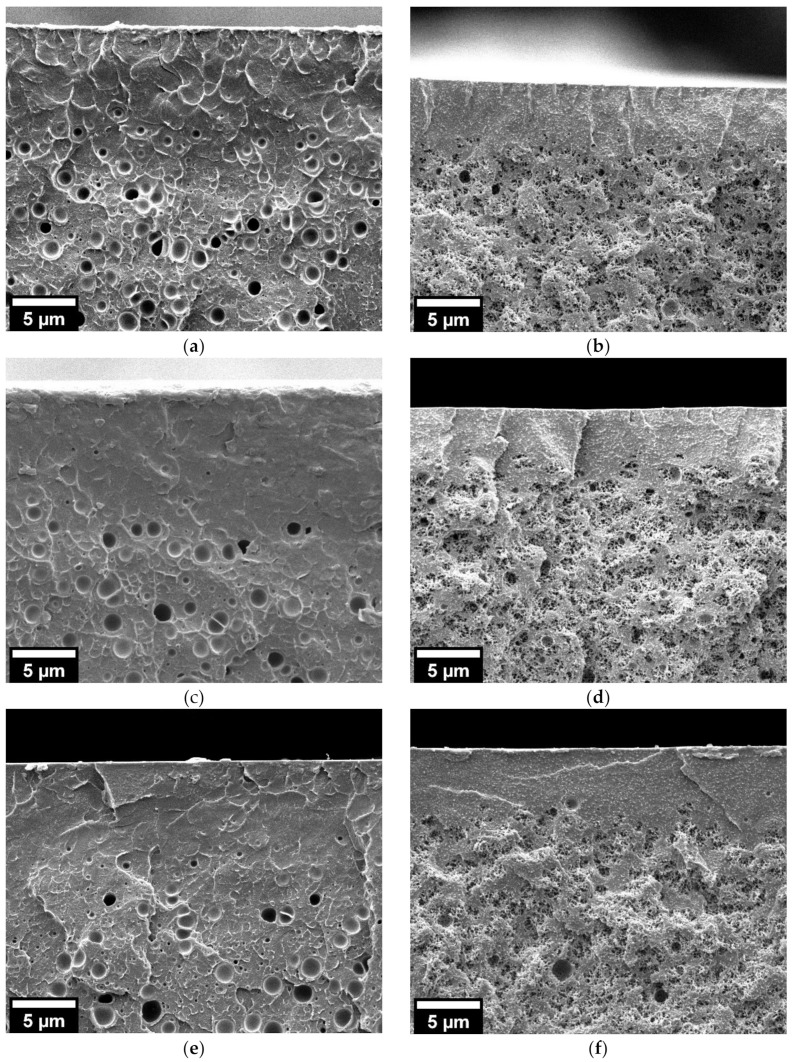
HRSEM micrographs of the solid outer layer of neat PMMA (left) and 25/75 PMMA/MAM (right) foams obtained from films at saturation pressure and temperature of 30 MPa and 50 °C, respectively, and a post-foaming procedure carried out for 1 min at different temperatures (**a**,**b**) 40 °C; (**c**,**d**) 60 °C; (**e**,**f**) 80 °C.

The influence of the post-foaming process on this feature was also studied. For bulk neat PMMA samples, the post-foaming temperature presents a noticeable influence on the solid outer layers, decreasing their thickness from about 140 nm to 9 µm (Table 2 and Figure S7, see Supplementary Materials). However, the solid outer layers of neat PMMA films did not present changes due to the post-foaming process. In this case the effect of the foaming mold was predominant in the foaming process (as explained previously when discussing the cellular structure) (Table 2 and Figure 6). Both bulk and film samples of 25/75 PMMA/MAM blends also kept the same behavior, with no influence of the post-foaming process on the solid outer layers (Table 2, Figure 6 and Figure S7, see Supplementary Materials). In addition, it can be appreciated that the transition from the solid skin to a homogeneous foamed core is abrupt in the case of 25/75 PMMA/MAM films, while it seems progressive for neat PMMA films (Figure 6). This behavior agrees with the diverse nucleation mechanism in these samples, heterogeneous in 25/75 PMMA/MAM and homogeneous in neat PMMA [39].

Therefore, it is confirmed that the nanostructuration of 25/75 PMMA/MAM blends, both out-of-equilibrium and near-equilibrium, controls the formation of the solid outer layers, being able to provide thin layers independently of the foaming parameters. Moreover, the use of the foaming mold, although necessary to foam the films, does not provide an additional decrease in these solid layers. However, as the foaming mold seems to hinder any influence of the post-foaming even in neat PMMA samples, it could be expected that further developments on the foaming of films, avoiding or decreasing the expansion limitation, would help to decrease the solid outer layers.

**Table 1:** Main characteristics of the neat PMMA and 25/75 PMMA foams obtained in a one-step foaming process. Histograms of the cell size distribution of each sample can be found in Figure S8, see Supplementary Materials.

Sample	Geometry	Saturation Pressure (MPa)	Saturation Temperature (°C)	Relative Density Average/SD		Cell Size (nm) Average/SD		Solid Skin Thickness (µm) Average/SD	
PMMA	Bulk	20	40	0.78	0.03	1230	960	267.4	23.4
PMMA	Bulk	20	50	0.80	0.02	1310	703	250.5	36.2
PMMA	Bulk	20	60	0.81	0.03	1945	872	241.1	16.0
PMMA	Bulk	30	40	0.66	0.04	325	128	177.4	19.4
PMMA	Bulk	30	50	0.67	0.03	650	372	136.9	12.6
PMMA	Bulk	30	60	0.62	0.05	1745	740	88.3	9.2
PMMA	Film	20	40	-	-	-	-	-	-
PMMA	Film	20	50	-	-	833	262	14.9	1.6
PMMA	Film	20	60	-	-	1419	432	10.4	2.3
PMMA	Film	30	40	-	-	652	511	4.9	1.1
PMMA	Film	30	50	-	-	972	467	8.2	1.5
PMMA	Film	30	60	-	-	1296	560	8.1	1.8
25/75 PMMA/MAM	Bulk	20	40	0.71	0.04	100	31	29.2	4.4
25/75 PMMA/MAM	Bulk	20	50	0.68	0.05	110	39	10.7	1.0
25/75 PMMA/MAM	Bulk	20	60	0.65	0.04	120	65	8.4	1.7
25/75 PMMA/MAM	Bulk	30	40	0.66	0.03	135	60	9.4	1.3
25/75 PMMA/MAM	Bulk	30	50	0.69	0.04	116	48	4.3	1.4
25/75 PMMA/MAM	Bulk	30	60	0.59	0.03	140	78	7.2	1.7
25/75 PMMA/MAM	Film	20	40	-	-	96	27	11.3	1.9
25/75 PMMA/MAM	Film	20	50	-	-	124	45	7.4	1.0
25/75 PMMA/MAM	Film	20	60	-	-	210	110	6.9	1.3
25/75 PMMA/MAM	Film	30	40	-	-	95	47	6.5	0.4
25/75 PMMA/MAM	Film	30	50	-	-	162	74	4.8	0.9
25/75 PMMA/MAM	Film	30	60	-	-	258	164	3.3	0.7

**Table 2:** Main characteristics of the neat PMMA and 25/75 PMMA foams obtained in a two-step foaming process. Histograms of the cell size distribution of each sample can be found in Figure S9, see Supplementary Materials.

Sample	Geometry	Post-Foaming Temperature (°C)	Post-Foaming Time (min)	Relative Density Average/SD		Cell Size (nm) Average/SD		Solid Skin Thickness (µm) Average/SD	
PMMA	Bulk	-	-	0.67	0.03	650	372	136.9	12.6
PMMA	Bulk	40	1	0.51	0.05	1675	1138	61.9	6.5
PMMA	Bulk	60	1	0.38	0.04	1685	1385	24.0	2.0
PMMA	Bulk	80	1	0.26	0.05	1800	1487	9.1	1.7
PMMA	Film	-	-	-		972	467	8.2	1.5
PMMA	Film	40	1	-		1250	436	7.9	0.7
PMMA	Film	60	1	-		1446	404	10.6	1.5
PMMA	Film	80	1	-		1191	360	9.7	1.7
25/75 PMMA/MAM	Bulk	-	-	0.69	0.04	116	48	4.3	1.4
25/75 PMMA/MAM	Bulk	40	1	0.67	0.03	123	51	7.0	1.5
25/75 PMMA/MAM	Bulk	60	1	0.67	0.03	103	49	8.9	1.1
25/75 PMMA/MAM	Bulk	80	1	0.68	0.04	105	49	9.8	2.0
25/75 PMMA/MAM	Film	-	-	-		162	74	4.8	0.9
25/75 PMMA/MAM	Film	40	1	-		141	63	5.6	0.4
25/75 PMMA/MAM	Film	60	1	-		136	62	3.7	0.6
25/75 PMMA/MAM	Film	80	1	-		118	61	4.8	0.8

### 3.4. Cell Nucleation in the Near-Equilibrium Nanostructuration of PMMA/MAM Blends

The near-equilibrium nanostructuration of 90/10 PMMA/MAM blends was not suitable for the production of nanocellular foams, instead leading to microcellular ones. However, it provided a unique opportunity to further study the cell nucleation in the nanostructuration. Recent theoretical considerations have proposed that the extraordinary nucleation efficiency of PMMA/MAM nanodomains is related to the nucleation process happening directly inside the PBA phase, instead of on the PMMA–PBA interphases [23]. Previous experimental evidence clearly shows the relationship between the nanodomains and the cell nucleation, but cannot confirm where exactly the nucleation is happening [28].

**Figure 7:**
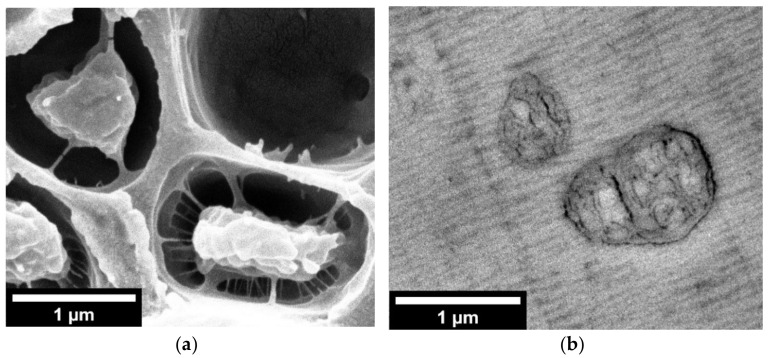
HRSEM micrograph of the 90/10 PMMA/MAM foams obtained from films at saturation pressure and temperature respectively of 30 MPa and 60 °C (**a**); TEM micrograph of the nanostructure of the 90/10 PMMA/MAM solid films (**b**).

On the contrary, the size of the near-equilibrium micelles of 90/10 PMMA/MAM films, and the quite low expansion achievable with the foaming mold, enables direct visual information on how the cells are formed in these materials (Figure 7a). In these near-equilibrium nanostructures, both the core and external diameter of the micelle present sizes of hundreds of nm, reaching about 1 µm in some cases (Figure 7b). HRSEM micrographs of the foamed 90/10 PMMA/MAM films show the remaining structure of the micelle (Figure 7a). The PMMA core remains intact in the center of the micelle, while the shell, initially composed of PBA, has expanded inside the surrounding PMMA matrix. Almost all the cells show this morphology (Figure S2, see Supplementary Materials), with the empty cells presenting remains of the filaments attaching the core (Figure 7a, top right cell), which was probably removed with the other half of the sample during the fracture procedure.

**Figure 8:**
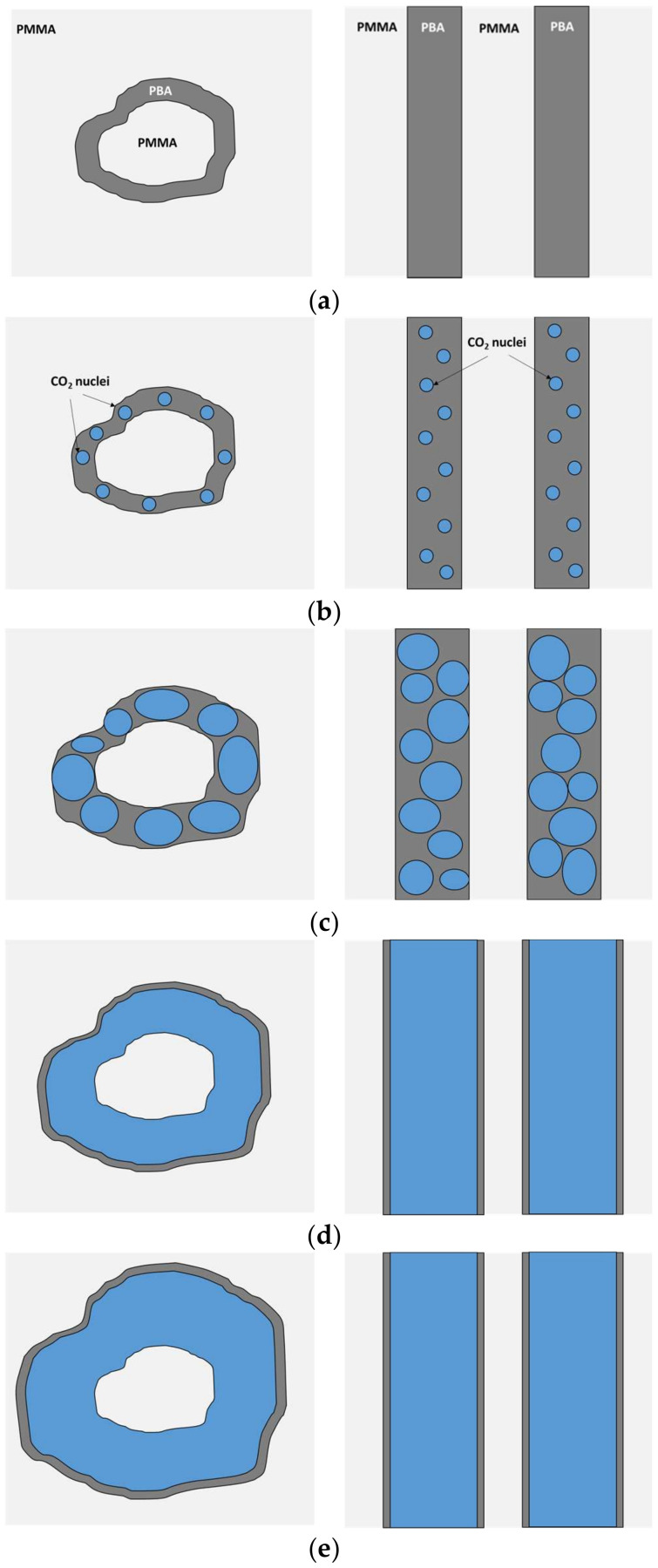
Scheme of the nucleation and cell growth process in the 90/10 (left) and 25/75 (right) PMMA/MAM nanostructures: nanostructure description (**a**); cell nucleation on the PBA phase (**b**); nuclei grow inside the PBA phase and expansion of the PMMA matrix surrounding the PBA phase (**c**); nuclei coalescence inside the PBA phase while the PMMA matrix keeps expanding (**d**); stabilization of the cellular structure (**e**). PMMA, PBA, and CO_2_ phases are depicted in light gray, dark gray, and blue, respectively.

The presence of the micelle cores almost perfectly centered in the cells (Figure 7 and Figure S2, see Supplementary Materials), and the proposed energetic preference of nucleation to happen directly inside the PBA phase (i.e., decreasing the energy barrier due to the lower Gibbs energy of the PBA phase and avoiding the contribution of the line tension) [23,28], suggest that the cell nucleation happens simultaneously at several points of the PBA shell (Figure 8a,b). Moreover, the remaining structures or filaments between the micelle cores and the cell walls could be considered evidence of multiple nucleation sites, as proposed in the work of Liu et al. [44]. Another nucleation process, such as a single nucleation point or nucleation in the interfaces, would probably displace the micelle cores from the center. Then, as the strength of the PBA phase is much lower than those of the surrounding PMMA, these nucleation points will quickly grow and coalescence, forming a gas-filled shell on the micelle while starting the expansion of the cell (Figure 8c,d). Finally, the expansion of the cell stops once the surrounding PMMA matrix is no longer plasticized or a balance of pressures has been reached (Figure 8e). Following this hypothesis, several nucleation points will be generated during the foaming process in 25/75 PMMA/MAM blends, both with near-equilibrium and out-of-equilibrium nanostructures (Figure 8a,b). Then, these nuclei will start to grow inside the PBA phase, inducing the expansion of the surrounding PMMA matrix, and quickly coalescence into a continuous gaseous phase (Figure 8c,d). However, in the case of co-continuous nanostructures, once this continuous gaseous phase is formed, the internal pressure can easily escape the sample through the open-cell structure, and no further expansion is possible (Figure 8e). It should be noticed that the cell nucleation, growth, and stabilization mechanisms proposed in Figure 8 explain the absence of changes in the post-foaming of the 25/75 PMMA/MAM foams. Both the out-of-equilibrium and near-equilibrium nanostructuration of bulk and film 25/75 PMMA/MAM samples are co-continuous (Figure 3, Figure 5 and Figure S4), and once the open-cell nanocellular structure is formed (in the first foaming step), the internal gas pressure, the driven force of the expansion, escaped through the cells. Moreover, this explanation is fully consistent with the previous evidence on the nucleation effect of PMMA/MAM nanodomains, as well as with the higher CO_2_ diffusivity out of the samples previously determined for neat MAM and PMMA/MAM blends with high MAM contents [28,45]. Focusing on dispersed nanodomains, an apparent contradiction to the proposed mechanism can be identified as nanocellular foams previously obtained from 90/10 PMMA/MAM out-of-equilibrium blends did not show remaining PMMA cores [28]. However, the presence or absence of these remaining cores is directly related to their composition. The cores of the out-of-equilibrium micelles are expected to be composed only of PMMA chains of the MAM molecules, while the near-equilibrium micelles should present homopolymer PMMA chains (Figure S10 and Section S5, see Supplementary Materials). Therefore, the PMMA core of the out-of-equilibrium micelles is pulled out (e.g., disentangled) during the nucleation and expansion happening in the PBA phase, while the homopolymer PMMA chains are barely affected in the near-equilibrium micelles (Figure S10 and Section S5, see Supplementary Materials). In addition, previous evidence of nucleation induced by dispersed MAM molecules (i.e., non-aggregated forming nanodomains) is fully consistent with the proposed negligible role of the PMMA–PBA interfaces in the nucleation process [42].

Taking into account the proposed foaming process on these samples, two strategies can be proposed for obtaining low density open-cell nanocellular PMMA/MAM foams. On the one hand, the use of high-efficient gas barriers, such as the metallic plates, but without constraining the macroscopic expansion could help to decrease the density by keeping the gas inside the open-cell structure for a longer time. On the other hand, adjusting the near-equilibrium or out-of-equilibrium PMMA/MAM nanostructures to obtain a higher volumetric percentage of co-continuous PBA phase, could provide foams with lower density, as the current evidence suggests that the available porosity will be at least equivalent to the PBA phase volume plus some additional expansion of the surrounding PMMA matrix.

## 4. Conclusions

The self-assembled nanostructuration of 90/10 PMMA/MAM blends has been found to be highly dependent on the fabrication conditions. On the one hand, out-of-equilibrium blends obtained by extrusion provide micelles with sizes of tens of nanometers, quite appropriate for the production of nanocellular foams. On the other hand, near-equilibrium blends obtained by solvent casting provide bigger micelles with sizes of hundreds of nanometers, and are unable to produce nanocellular foams. On the contrary, the self-assembled nanostructuration of 25/75 PMMA/MAM blends is more stable, showing less dependence on the fabrication conditions. Both out-of-equilibrium and near-equilibrium nanostructures are co-continuous, showing lamella thicknesses of a few tens of nanometers. Accordingly, the nanostructuration of 25/75 PMMA/MAM blends is suitable for the production of nanocellular foams, independent of the production route. This is a promising advantage of these materials for the potential development of continuous extrusion foaming approaches to produce nanocellular foams.

Moreover, the nanocellular structures obtained from bulk and film samples of 25/75 PMMA/MAM blends show comparable features, although the expansion of film samples is severely limited due to the required foaming mold, which also induces a significant cell coalescence at the higher saturation temperatures. The limitations induced by the foaming mold were also tested by using reference neat PMMA samples. Further expansion of the obtained foams was attempted by introducing a post-foaming stage at different temperatures. It was found that this approach was successful in neat PMMA bulk samples, but it did not have any effect either in 25/75 PMMA/MAM bulk samples or in neat PMMA or 25/75 PMMA/MAM films.

The influence of the nanostructuration on the presence of solid outer layers was also studied on the obtained open-cell nanocellular foams from 25/75 PMMA/MAM blends. It was found that the heterogeneous nucleation induced by the nanostructuration presents a major role in the reducing these solid outer layers, reaching thicknesses of about 5–10 µm independently of the foaming parameters. Similar thicknesses were only achievable by neat PMMA samples by optimizing the production parameters or using the foaming mold for films, being frequent to obtain thicknesses well over 100 µm in bulk samples for most of the foaming parameters employed.

Finally, the microcellular foams obtained from 90/10 PMMA/MAM films showing the near-equilibrium nanostructure, with micelles of hundreds of nanometers or even near one micron, provided the first direct evidence of the cell nucleation happening inside the PBA phase. In fact, the obtained cellular structure still showed the PMMA nucleus in the center of the cells, surrounded by an expanded gaseous shell grown inside the former PBA shell. From this evidence and previous experimental and theoretical results, it was possible to propose an updated scheme for the cell nucleation, growth, and stabilization on PMMA/MAM samples which explains the diverse behaviors found experimentally in this work.

## Data Availability

The data presented in this study are available in this article and its corresponding Supplementary Materials.

## Supplementary Materials

The following are available online at https://www.mdpi.com/article/10.3390/nano11112834/s1, Figure S1: TEM micrograph of the nanostructuration of a 90/10 PMMA/MAM near-equilibrium blend (a). Diameter histogram of the micelles of 90/10 PMMA/MAM near-equilibrium blends (b), Figure S2: Low magnification HRSEM micrograph of the 90/10 PMMA/MAM foams obtained from films at saturation pressure and temperature respectively of 30 MPa and 60 °C, Figure S3: HRSEM micrographs of the cellular structure of the neat PMMA foams obtained from bulk samples (left) and films (right) at 20 MPa and different saturation temperature: (a,b) 40 °C; (c,d) 50 °C; (e,f) 60 °C, Figure S4: HRSEM micrographs of the cellular structure of the 25/75 PMMA/MAM foams obtained from bulk samples (left) and films (right) at 20 MPa and different saturation temperature: (a,b) 40 °C; (c,d) 50 °C; (e,f) 60 °C, Figure S5: Low magnification HRSEM micrographs of the 25/75 PMMA/MAM foams obtained from bulk samples (left) and films (right) at 30 MPa and different saturation temperature: (a,b) 40 °C; (c,d) 50 °C; (e,f) 60 °C, Figure S6: Low magnification HRSEM micrographs of the 25/75 PMMA/MAM foams obtained from bulk samples (left) and films (right) at saturation pressure and temperature respectively of 30 MPa and 50 °C and a post-foaming carried during 1 min at different temperatures (a,b) 40 °C; (c,d) 60 °C; (e,f) 80 °C, Figure S7: HRSEM micrographs of the solid outer layer of neat PMMA (left) and 25/75 PMMA/MAM (right) foams obtained from bulk samples at saturation pressure and temperature respectively of 30 MPa and 50 °C and a post-foaming carried out for 1 min at different temperatures (a,b) 40 °C; (c,d) 60 °C; (e,f) 80 °C, Figure S8. Cell size histograms corresponding to the samples included in Table 1: PMMA bulk samples foamed at 20 MPa and 40 (a), 50 (b), or 60 (c) °C, 30 MPa and 40 (d), 50 (e), or 60 (f) °C; PMMA film samples foamed at 20 MPa and 50 (g), or 60 (h) °C, 30 MPa and 40 (i), 50 (j), or 60 (k) °C; 25/75 PMMA/MAM bulk samples foamed at 20 MPa and 40 (l), 50 (m), or 60 () °C, 30 MPa and 40 (o), 50 (p), or 60 (q) °C; 25/75 PMMA/MAM film samples foamed at 20 MPa and 40 (r), 50 (s), or 60 (t) °C, 30 MPa and 40 (u), 50 (v), or 60 (w) °C, Figure S9. Cell size histograms corresponding to the samples included in Table 2 (with the exception of samples also included in Table 1 and Figure S8): PMMA bulk samples post-foamed for 1 min at 40 (a), 60 (b), or 80 (c) °C; PMMA film samples post-foamed for 1 min at 40 (d), 60 (e), or 80 (f) °C; 25/75 PMMA/MAM bulk samples post-foamed for 1 min at 40 (g), 60 (h), or 80 (i) °C; 25/75 PMMA/MAM film samples post-foamed for 1 min at 40 (j), 60 (k), or 80 (l) °C, Figure S10. Scheme of the evolution of the nanostructures of 90/10 PMMA/MAM near-equilibrium (a) and out-of-equilibrium (b) blends during the foaming process: before (up), nucleation (middle), and stabilization (bottom).
