# Supplementary material for: Nanostructure of PMMA/MAM Blends Prepared by Out-of-Equilibrium (Extrusion) and Near-Equilibrium (Casting) Self-Assembly and Their Nanocellular or Microcellular Structure Obtained from CO2 Foaming
Source: Nanomaterials (Basel). 2021 Oct 25;11(11):2834. doi: 10.3390/nano11112834 (PMC8620990; doi:10.3390/nano11112834)
Supplement: Supplementary file 1 [file nanomaterials-11-02834-s001.zip › nanomaterials-1398362-supplementary.pdf]

Article

Nanostructure of PMMA/MAM blends prepared by Out-of-Equilibrium (Extrusion) and Near-Equilibrium (Casting) Self-Assembly and their Nanocellular or Microcellular Structure obtained from CO_2 foaming

Suset Barroso-Solares [1,2]*, Victoria Bernardo [3], Daniel Cuadra-Rodriguez [2] and Javier Pinto [1,2]*

[1] BioEcoUVA Research Institute on Bioeconomy, University of Valladolid, 47011 Valladolid, Spain
[2] Cellular Materials Laboratory (CellMat), Condensed Matter Physics Department, University of Valladolid, 47011 Valladolid, Spain; dcuadra@fmc.uva.es
[3] CellMat Technologies S.L., Paseo de Belen 9-A (CTTA Building), 47011 Valladolid, Spain; v.bernardo@cellmattechnologies.com
* Correspondence: sbarroso@fmc.uva.es (S.B.-S.); jpinto@fmc.uva.es. (J.P.)

Supplementary Materials

S.1. Out-of-equilibrium and near-equilibrium nanostructuration of PMMA/MAM blends

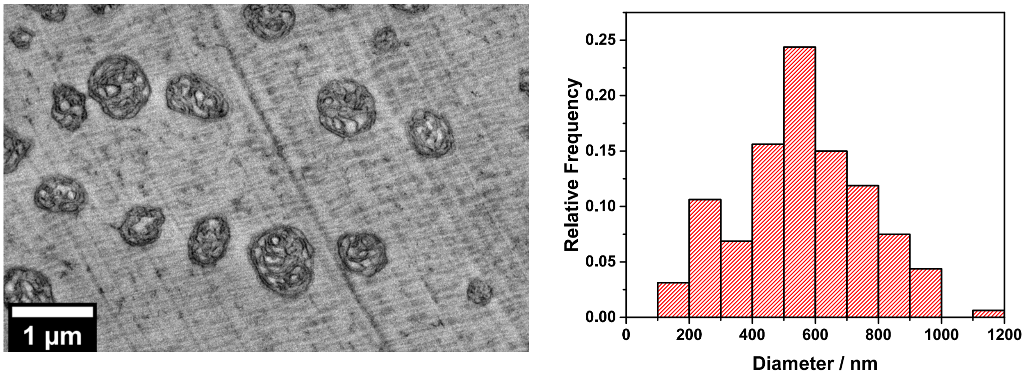

(a) (b)

Figure S1. TEM micrograph of the nanostructuration of a 90/10 PMMA/MAM near-equilibrium blend (**a**). Diameter histogram of the micelles of 90/10 PMMA/MAM near-equilibrium blends (**b**).

S.2. Nanocellular foams obtained from out-of-equilibrium and near-equilibrium nanostructuration of PMMA/MAM blends

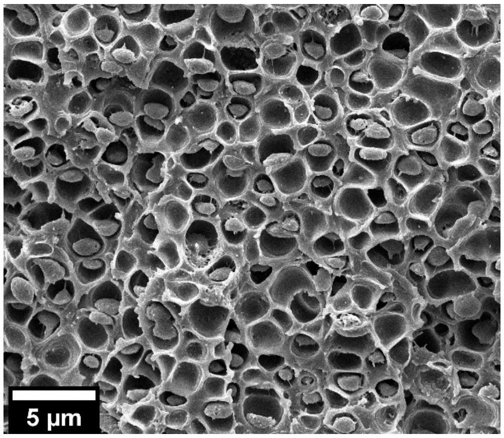

Figure S2. Low magnification HRSEM micrograph of the 90/10 PMMA/MAM foams obtained from films at saturation pressure and temperature respectively of 30 MPa and 60 °C. Similar microcellular structures are obtained by varying the saturation pressure and temperature.

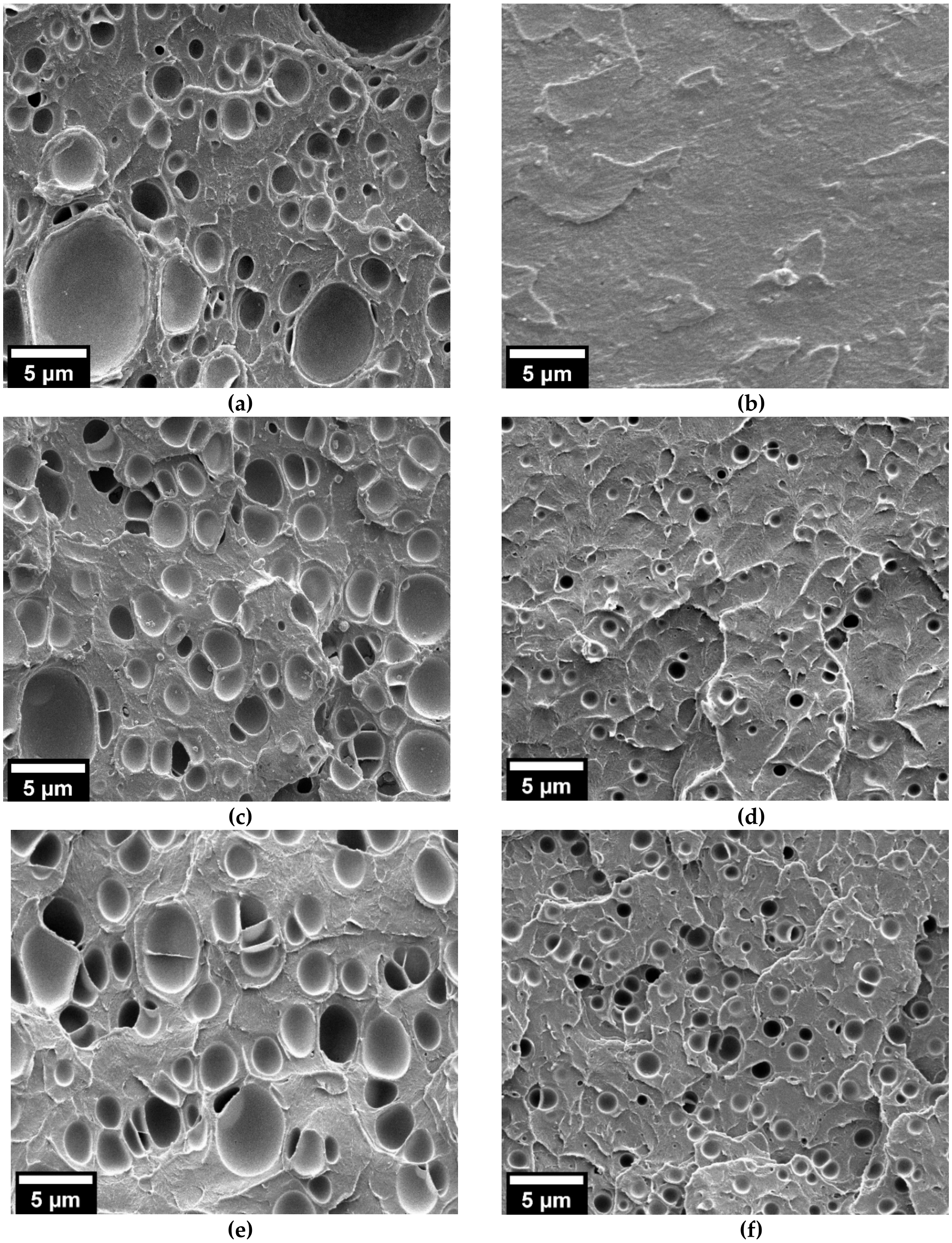

Figure S3. HRSEM micrographs of the cellular structure of the neat PMMA foams obtained from bulk samples (left) and films (right) at 20 MPa and different saturation temperature: (**a, b**) 40 °C; (**c, d**) 50 °C; (**e, f**) 60 °C.

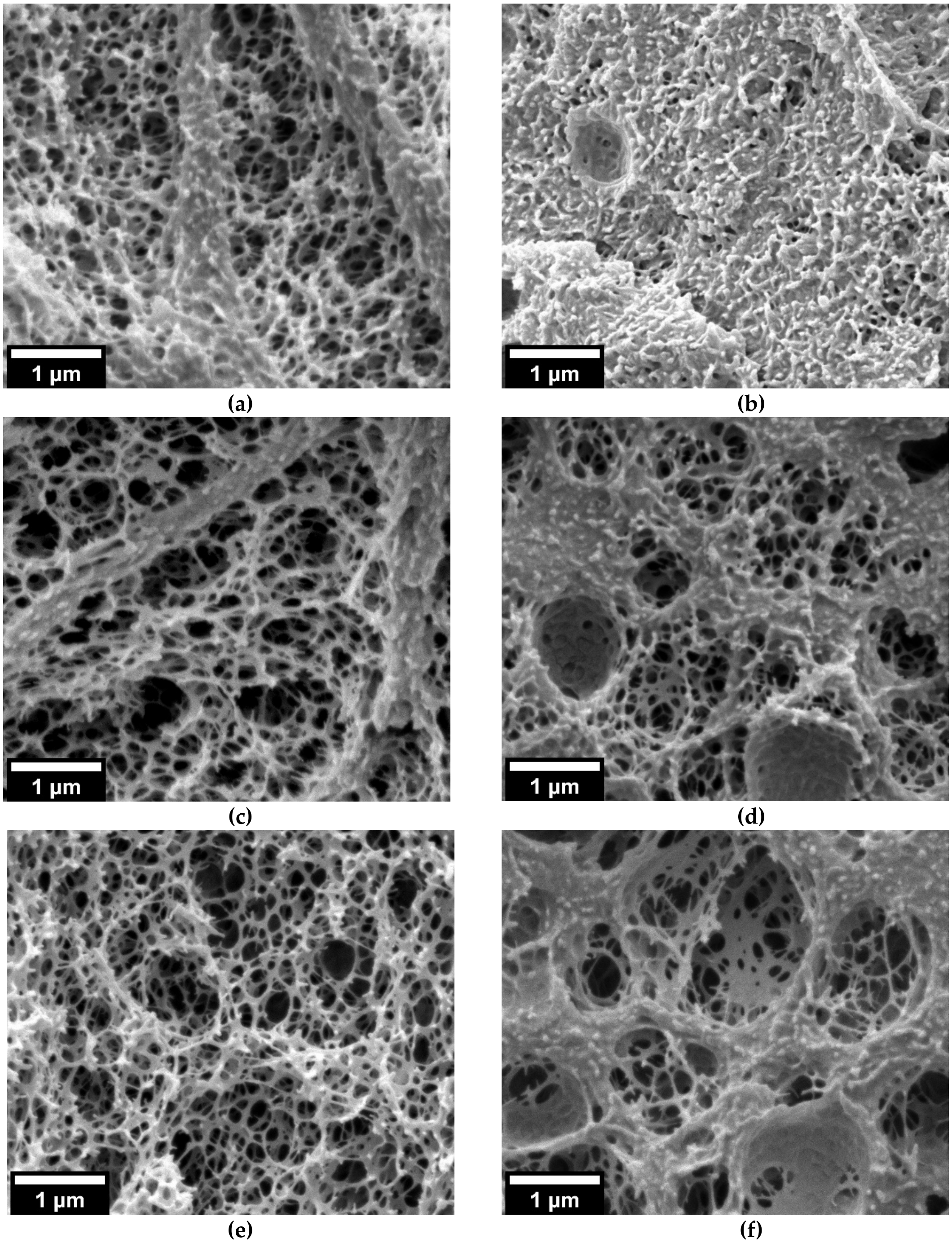

Figure S4. HRSEM micrographs of the cellular structure of the 25/75 PMMA/MAM foams obtained from bulk samples (left) and films (right) at 20 MPa and different saturation temperature: (**a, b**) 40 °C; (**c, d**) 50 °C; (**e, f**) 60 °C.

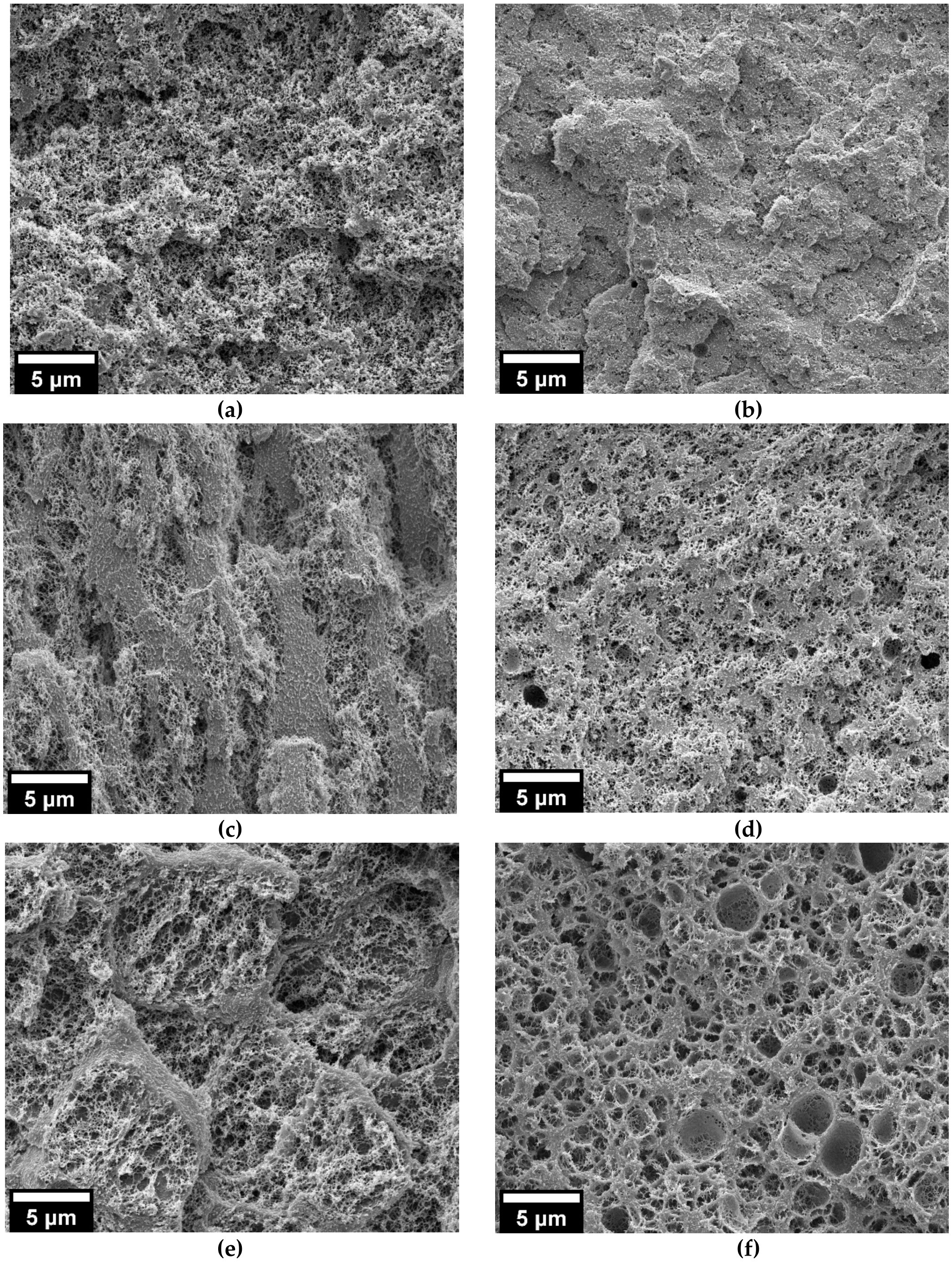

Figure S5. Low magnification HRSEM micrographs of the 25/75 PMMA/MAM foams obtained from bulk samples (left) and films (right) at 30 MPa and different saturation temperature: (**a, b**) 40 °C; (**c, d**) 50 °C; (**e, f**) 60 °C.

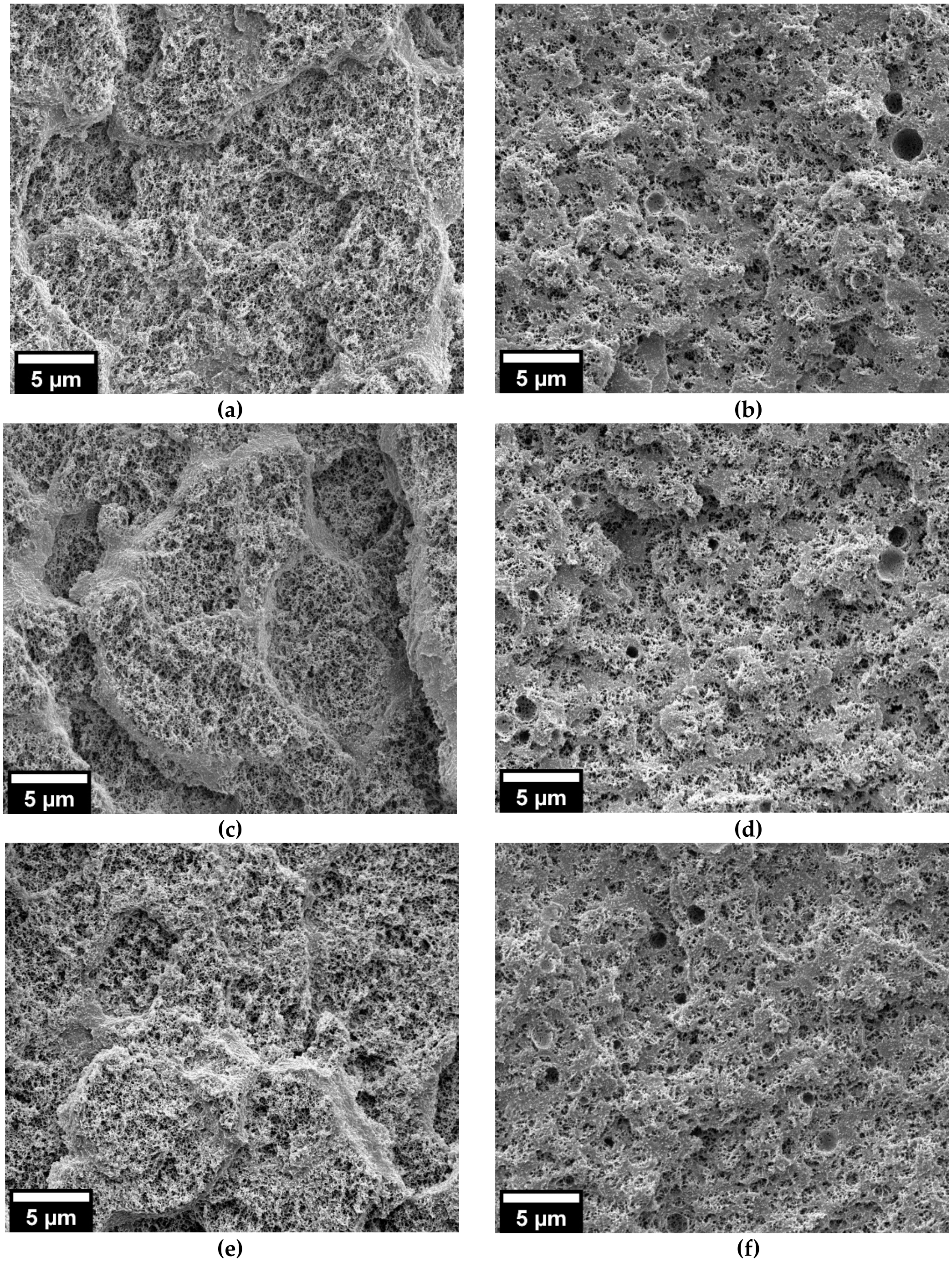

Figure S6. Low magnification HRSEM micrographs of the 25/75 PMMA/MAM foams obtained from bulk samples (left) and films (right) at saturation pressure and temperature respectively of 30 MPa and 50 °C and a post-foaming carried during 1 minute at different temperatures (**a, b**) 40 °C; (**c, d**) 60 °C; (**e, f**) 80 °C.

S.3 Influence of the co-continuous out-of-equilibrium and near-equilibrium nanostructuration of 25/75 PMMA/MAM blends on the solid outer layers

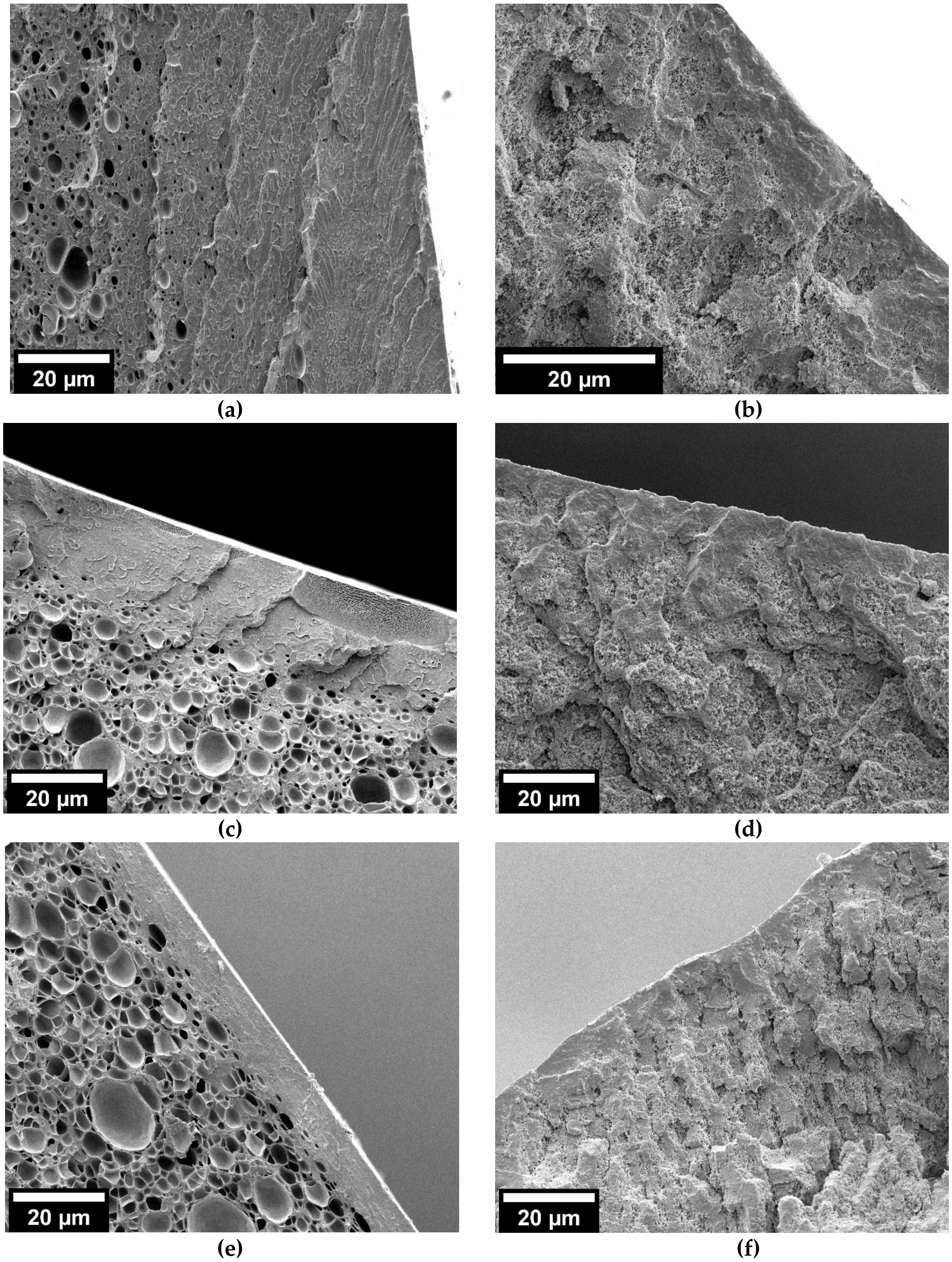

Figure S7. HRSEM micrographs of the solid outer layer of neat PMMA (left) and 25/75 PMMA/MAM (right) foams obtained from bulk samples at saturation pressure and temperature respectively of 30 MPa and 50 °C and a post-foaming carried during 1 minute at different temperatures (**a, b**) 40 °C; (**c, d**) 60 °C; (**e, f**) 80 °C.

S.4 Cell size distribution of the obtained foams

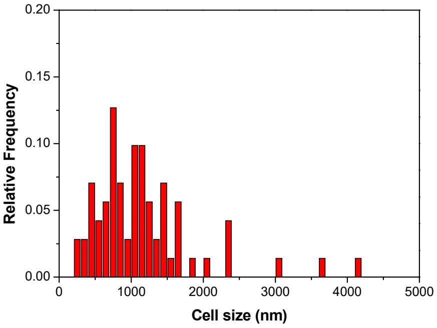

(a)

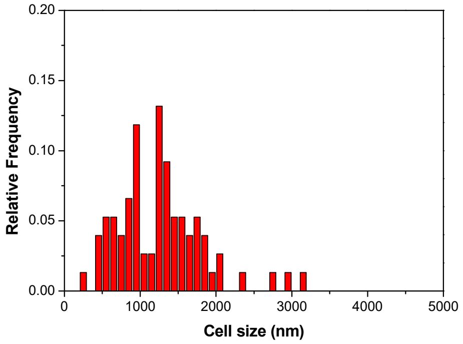

(b)

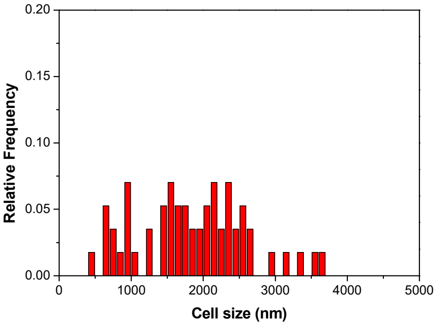

(c)

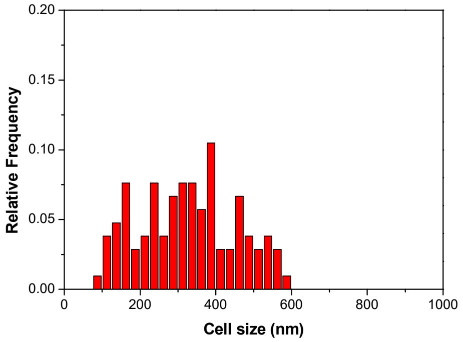

(d)

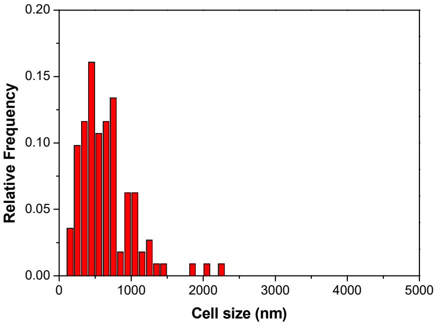

(e)

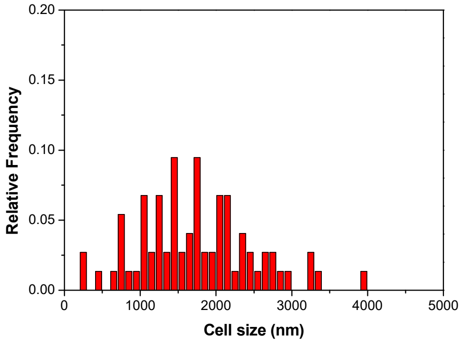

(f)

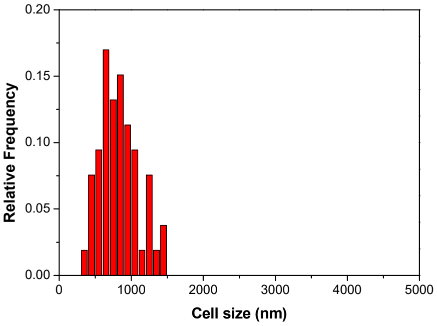

(g)

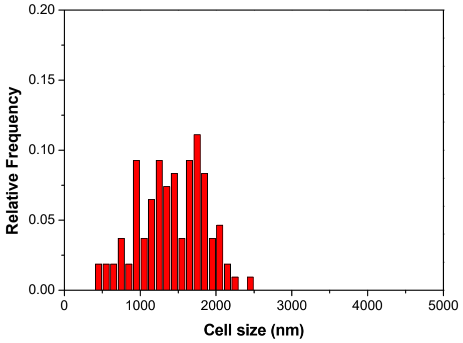

(h)

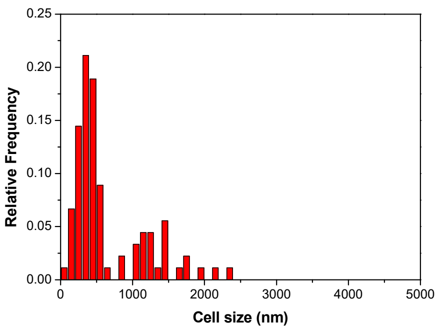
Relative Frequency
0.00
0.05
0.10
0.15
0.20
0.25
Cell size (nm)
0
1000
2000
3000
4000
5000

(i)

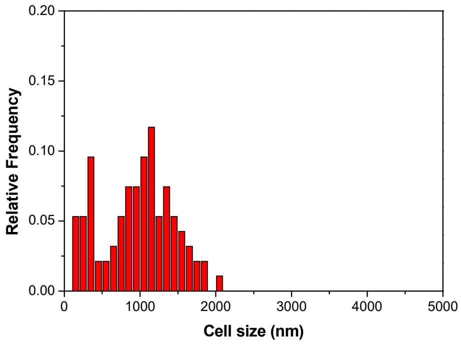
Relative Frequency
0.00
0.05
0.10
0.15
0.20
Cell size (nm)
0
1000
2000
3000
4000
5000

(j)

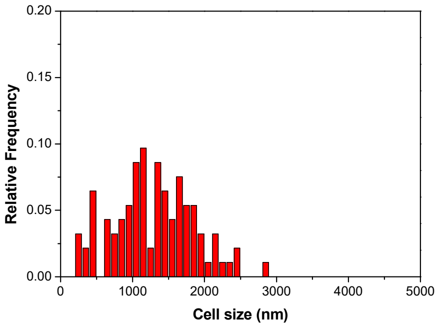
Relative Frequency
0.00
0.05
0.10
0.15
0.20
Cell size (nm)
0
1000
2000
3000
4000
5000

(k)

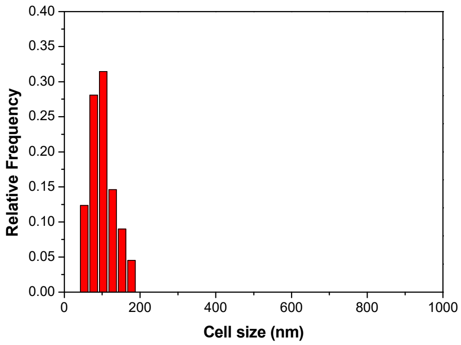
Relative Frequency
0.00
0.05
0.10
0.15
0.20
0.25
0.30
0.35
0.40
Cell size (nm)
0
200
400
600
800
1000

(l)

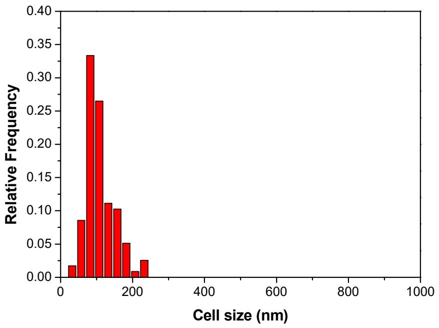
Relative Frequency
0.00
0.05
0.10
0.15
0.20
0.25
0.30
0.35
0.40
Cell size (nm)
0
200
400
600
800
1000

(m)

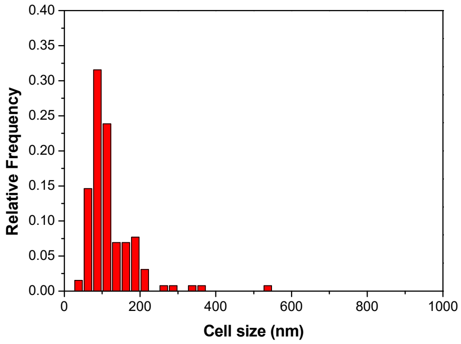
Relative Frequency
0.00
0.05
0.10
0.15
0.20
0.25
0.30
0.35
0.40
Cell size (nm)
0
200
400
600
800
1000

(n)

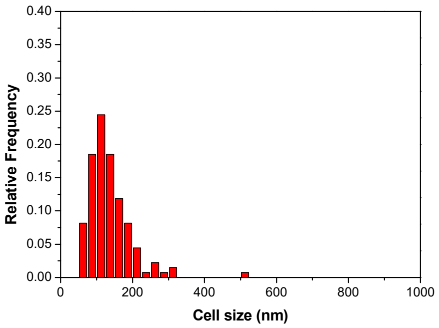
Relative Frequency
0.00
0.05
0.10
0.15
0.20
0.25
0.30
0.35
0.40
Cell size (nm)
0
200
400
600
800
1000

(o)

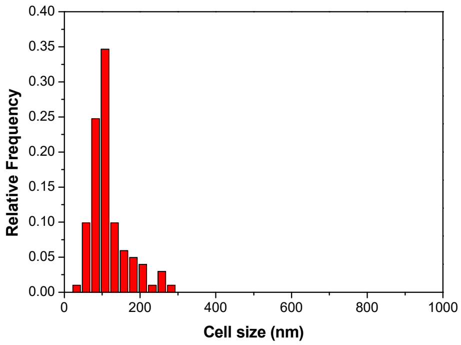
Relative Frequency
0.00
0.05
0.10
0.15
0.20
0.25
0.30
0.35
0.40
Cell size (nm)
0
200
400
600
800
1000

(p)

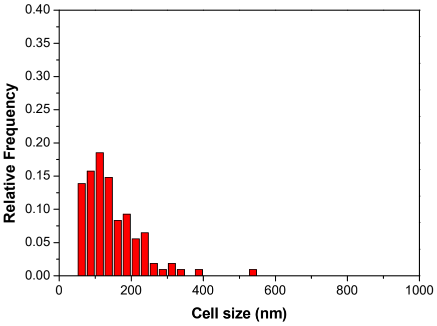
Relative Frequency
0.00 0.05 0.10 0.15 0.20 0.25 0.30 0.35 0.40
0 200 400 600 800 1000
Cell size (nm)

(q)

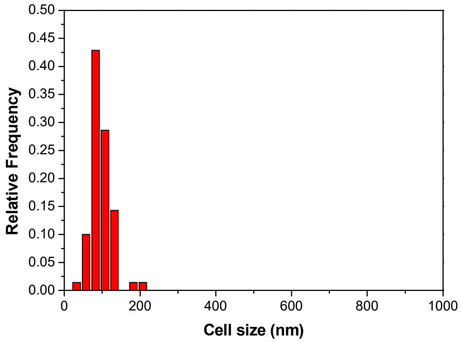
Relative Frequency
0.00 0.05 0.10 0.15 0.20 0.25 0.30 0.35 0.40 0.45 0.50
0 200 400 600 800 1000
Cell size (nm)

(r)

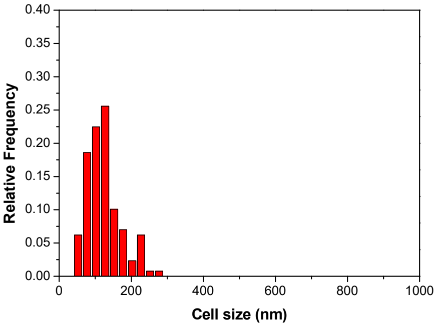
Relative Frequency
0.00 0.05 0.10 0.15 0.20 0.25 0.30 0.35 0.40
0 200 400 600 800 1000
Cell size (nm)

(s)

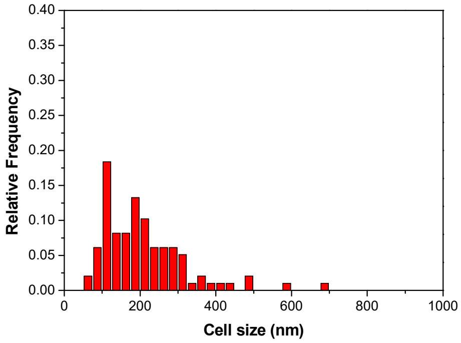
Relative Frequency
0.00 0.05 0.10 0.15 0.20 0.25 0.30 0.35 0.40
0 200 400 600 800 1000
Cell size (nm)

(t)

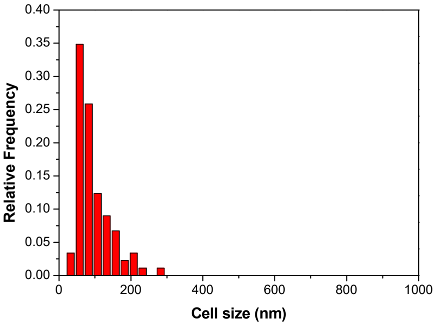
Relative Frequency
0.00 0.05 0.10 0.15 0.20 0.25 0.30 0.35 0.40
0 200 400 600 800 1000
Cell size (nm)

(u)

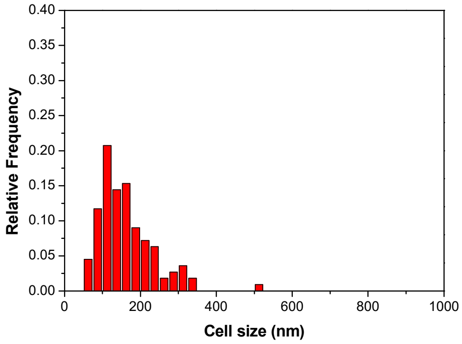
Relative Frequency
0.00 0.05 0.10 0.15 0.20 0.25 0.30 0.35 0.40
0 200 400 600 800 1000
Cell size (nm)

(v)

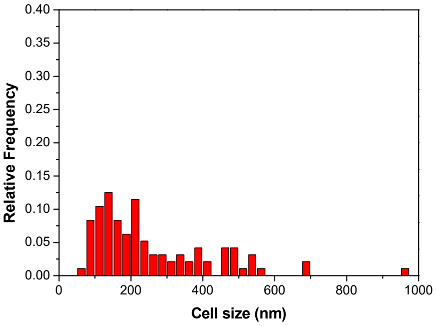
Relative Frequency
0.00 0.05 0.10 0.15 0.20 0.25 0.30 0.35 0.40
0 200 400 600 800 1000
Cell size (nm)

(w)

Figure S8. Cell size histograms corresponding to the samples included in Table 1: PMMA bulk samples foamed at 20 MPa and 40 (a), 50 (b), or 60 (c) °C, 30 MPa and 40 (d), 50 (e), or 60 (f) °C; PMMA film samples foamed at 20 MPa and 50 (g), or 60 (h) °C, 30 MPa and 40 (i), 50 (j), or 60 (k) °C; 25/75 PMMA/MAM bulk samples foamed at 20 MPa and 40 (l), 50 (m), or 60 () °C, 30 MPa and 40 (o), 50 (p), or 60 (q) °C; 25/75 PMMA/MAM film samples foamed at 20 MPa and 40 (r), 50 (s), or 60 (t) °C, 30 MPa and 40 (u), 50 (v), or 60 (w) °C.

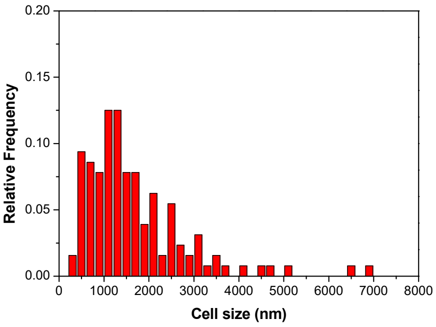

(a)

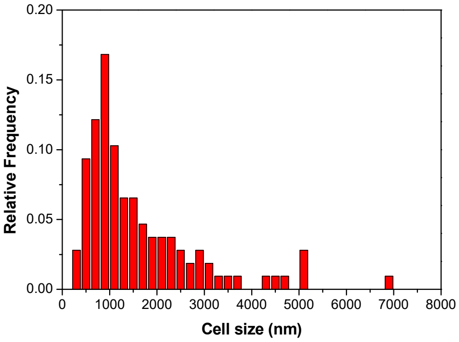

(b)

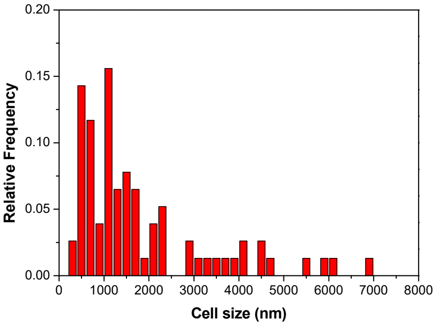

(c)

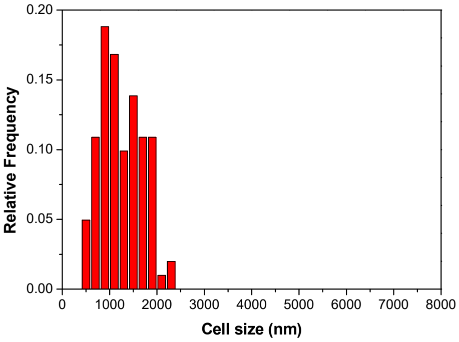

(d)

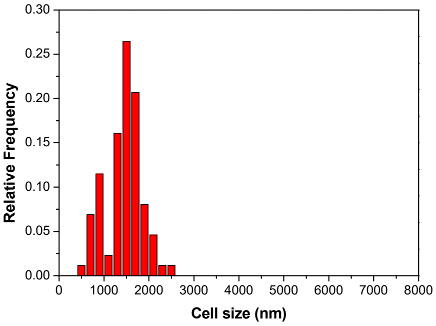

(e)

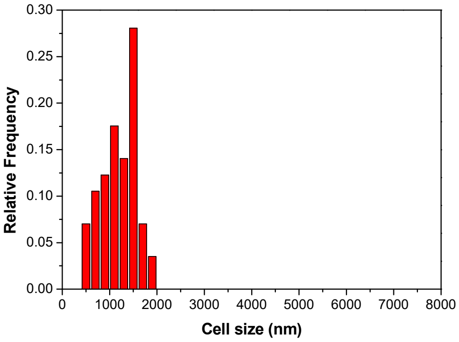

(f)

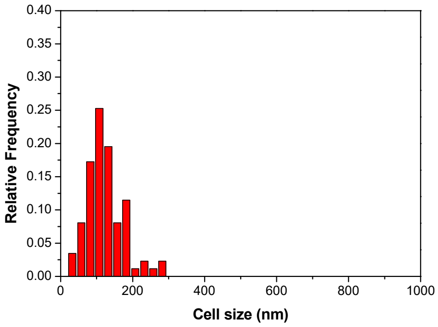

(g)

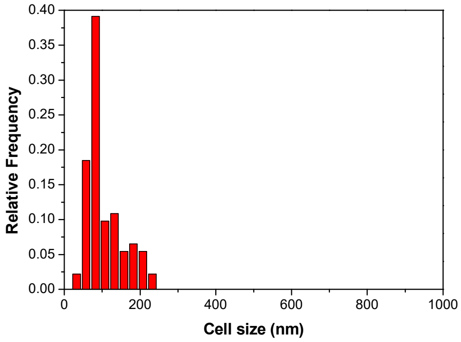

(h)

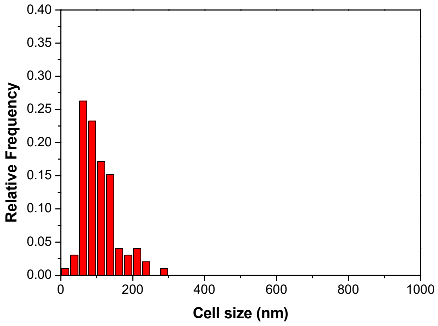

(i)

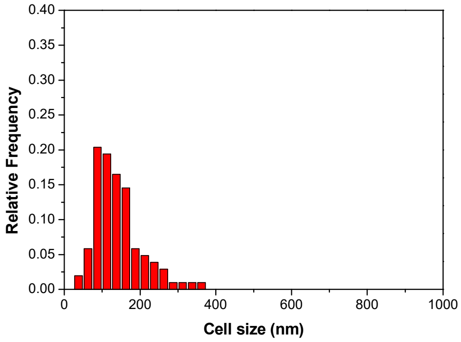

(j)

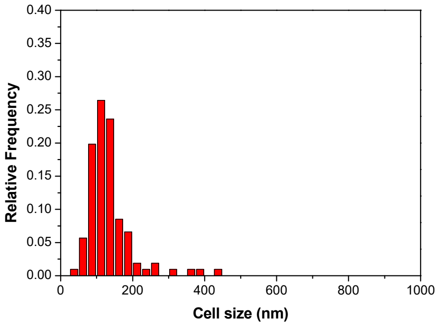

(k)

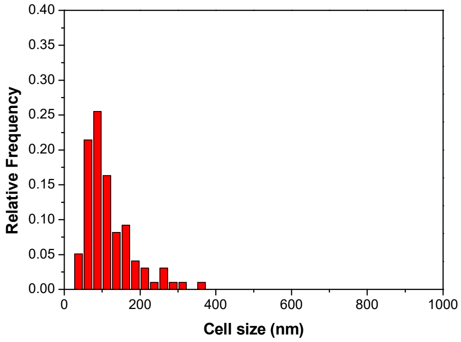

(l)

Figure S9. Cell size histograms corresponding to the samples included in Table 2 (with the exception of samples also included in Table 1 and Figure S.8): PMMA bulk samples post-foamed for 1 minute at 40 (a), 60 (b), or 80 (c) °C; PMMA film samples post-foamed for 1 minute at 40 (d), 60 (e), or 80 (f) °C; 25/75 PMMA/MAM bulk samples post-foamed for 1 minute at 40 (g), 60 (h), or 80 (i) °C; 25/75 PMMA/MAM film samples post-foamed for 1 minute at 40 (j), 60 (k), or 80 (l) °C.

S.5 Cell nucleation on the near-equilibrium nanostructuration of PMMA/MAM blends

First, the estimated dimensions of the PMMA chains of the MAM block copolymer should be taken into account. These chains have a M_n of about 27000 g/mol, while the MMA monomer has a M_0 of about 100.12 g/mol. Accordingly, the degree of polymerization ($N=M_n/M_0$) of the PMMA chains is about 269.68.

It is possible to estimate the potential maximum length of a stretched polymer chain using equation 1:

$$L = N \cdot d \cdot \sin\frac{\theta}{2} \quad [1]$$

Where d is the C-C bond length (about 0.154 nm) and θ the angle between adjacent C-C bonds of the PMMA backbone (about 109°). Accordingly, the maximum length of the PMMA chains of the MAM copolymers should be about 33.8 nm- Although this is an unrealistic magnitude for the polymer chain, it pointed out that the core of the near-equilibrium 90/10 PMMA/MAM micelles (> 100 nm) cannot be composed only by PMMA chains corresponding to MAM molecules (i.e., even unrealistic completely stretched PMMA chains cannot fulfil the core) (Figure S8).

In addition, an estimation of the PMMA chains volume can be obtained calculating their radius of gyration, which for PMMA can be obtained using equation 2:[1]

$$R_g = (0.096 \cdot M_w^{0.98})^{\frac{1}{2}} \quad [2]$$

Where M_w can be estimated taking into account the I_p of the MAM molecule (about 2).

The obtained radius of gyration of the PMMA chains is about 6.4 nm, which is of the same order of magnitude as the core of the 90/10 PMMA/MAM out-of-equilibrium micelles (about 10-15 nm).[2] Accordingly, it can be expected that the PMMA core of these micelles is composed only of PMMA chains of MAM molecules (Figure S8).

As a consequence, during the foaming process, the expansion of the PBA shell will induce the disentanglement of the PMMA core of out-of-equilibrium micelles, as the PMMA chains of the core are bonded to the PBA chains suffering the nucleation and expansion of the cell (Figure S8). On the contrary, the homopolymer PMMA chains present inside the core of 90/10 PMMA/MAM near-equilibrium micelles are not pulled out by the MAM molecules, due to the absence of chemical bonding, leading to a remaining solid core in the center of the final cells (Figure S8).

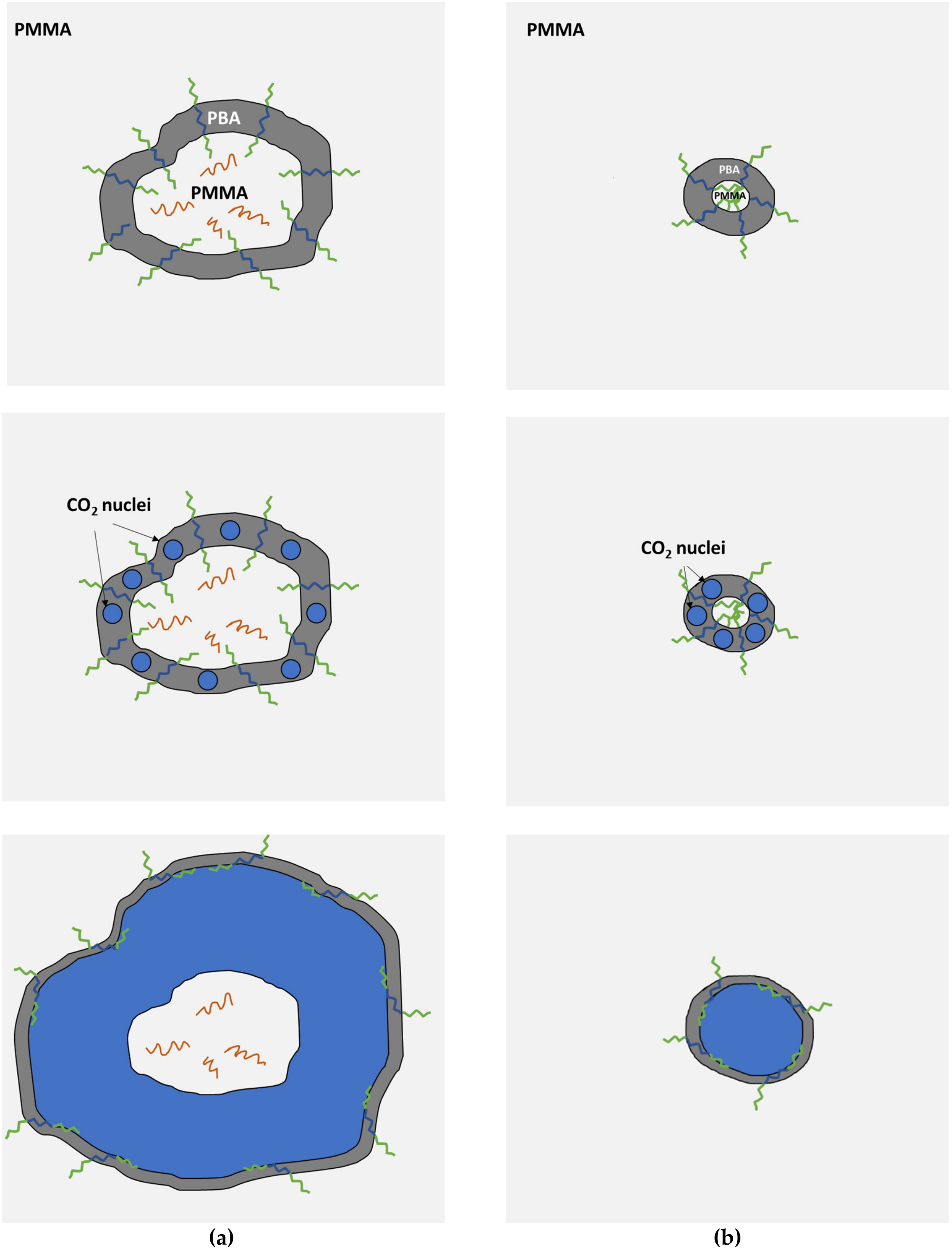

Figure S10. Scheme of the evolution of the nanostructures of 90/10 PMMA/MAM near-equilibrium (**a**) and out-of-equilibrium (**b**) blends during the foaming process: before (up), nucleation (middle), and stabilization (bottom). Homopolymer PMMA chains are shown in orange, while PMMA and PBA chains of the MAM molecules are respectively shown in green and blue. It should be noticed that this is a schematic representation, therefore the depicted polymer chains are not intended to be at exact scale.

References

1. Kirste, R.G.; Kruse, W.A.; Ibel, K. Determination of the conformation of polymers in the amorphous solid state and in concentrated solution by neutron diffraction. *Polymer (Guildf).* **1975**, *16*, 120–124.

2. Pinto, J.; Dumon, M.; Pedros, M.; Reglero, J.; Rodriguez-Perez, M.A. Nanocellular CO2 foaming of PMMA assisted by block copolymer nanostructuration. *Chem. Eng. J.* **2014**, *243*, 428–435.
